# Associations of circulating immunomarkers with the efficacy of immunotherapy for primary hepatic carcinoma

**DOI:** 10.1002/cam4.6754

**Published:** 2023-12-06

**Authors:** Sha Liu, Wentao Xu, Hang Shu, Ying Dai, Yingying Du, Yunmei Liu, Lei Huang, Guoping Sun

**Affiliations:** ^1^ Department of Oncology The First Affiliated Hospital of Anhui Medical University Hefei Anhui China; ^2^ School of Clinical Medicine Anhui Medical University Hefei Anhui China; ^3^ School of Cultural Heritage and Information Management Shanghai University Shanghai China; ^4^ Department of Oncology, Ruijin Hospital Shanghai Jiao Tong University School of Medicine Shanghai China; ^5^ Medical Center on Aging of Ruijin Hospital, MCARJH Shanghai Jiaotong University School of Medicine Shanghai China

**Keywords:** immunotherapy, liver cancer, peripheral blood cells, prognosis, treatment response

## Abstract

**Background:**

Peripheral blood immunomarkers are associated with prognosis in patients with solid tumors receiving chemotherapy or immunotherapy. In this study, the associations of circulating neutrophil‐to‐lymphocyte ratio (NLR), monocyte‐to‐lymphocyte ratio (MLR), and platelet‐to‐lymphocyte ratio (PLR), as well as their dynamic changes were investigated in relation to the efficacy of immunotherapy in patients with primary liver cancer.

**Methods:**

Comparisons were made between NLR, MLR, and PLR among individuals exhibiting disease control (defined as the best response of partial response [PR] or stable disease [SD]) and those with progressive disease (PD). Additionally, disease control rate (DCR), overall survival (OS), and progression‐free survival (PFS) were compared between individuals with different NLR, MLR, and PLR levels before initiating palliative immunotherapy. Furthermore, comparisons were made between patients with different alterations in the ratios at the second cycle of immunotherapy compared to baseline. These analyses were performed using univariate and multivariate approaches. A total of 119 Chinese patients with liver cancer who underwent immunotherapy were included in this study, which focused on hepatocellular carcinoma (HCC).

**Results:**

In cases with HCC (*n* = 104), the cutoffs of NLR, MLR, and PLR to differentiate treatment responders from nonresponders were 3.38, 0.28, and 227.18, respectively. Patients with the best response of PR or SD had significantly lower NLR and MLR. Patients with NLR <3.38 and those with MLR <0.28 significantly had longer OS and PFS than their counterparts, and those with PLR <227.18 had significantly longer PFS, both in overall patients and in various patient subgroups. Lower NLR, MLR, or PLR was associated with earlier BCLC stage, fewer metastatic sites, less frequent extrahepatic metastasis, or better performance status. For individuals who had an unfavorable baseline NLR ≥3.38, MLR ≥0.28, or a favorable baseline PLR <227.18 prior to first immunotherapy, a decrease in NLR, MLR, or PLR at Cycle 2 of immunotherapy was significantly associated with a higher DCR.

**Conclusions:**

Among patients with HCC who received immunotherapy, lower NLR, and MLR at baseline in overall patients were significantly associated with better disease control and more favorable survival outcomes (both OS and PFS), and lower PLR was significantly associated with longer PFS. The findings of this research may offer useful hints foranoptimized selection of patients with liver cancer who may benefit more from immunotherapy.

## Graphical abstract

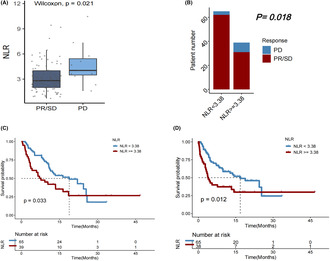

## 1 INTRODUCTION

Among all types of cancers, primary liver cancer has the sixth‐highest incidence rate and the fourth‐highest mortality rate.^1^, ^2^, ^3^, ^4^ The main pathological type of primary liver cancer is hepatocellular carcinoma (HCC; 85%–90%), and the other few cases are intrahepatic cholangiocarcinoma (ICC) and mixed types of HCC and ICC. Primary treatment modalities for individuals with liver cancer include surgical resection, radiofrequency ablation, transcatheter arterial chemoembolization, and tyrosine kinase inhibitors (TKIs) such as sorafenib and lenvatinib.^5^ Notably, the overall prognosis remains unsatisfactory.^6^

Chronic inflammation is a leading causal factor in many cases of HCC, with major underlying contributors including viral hepatitis, alcohol use, and nonalcoholic steatohepatitis. Chronic inflammation can be defined as the prolonged existence of an inefficient immune response, which is linked to the formation of an immunosuppressive environment characterized by elevated expression levels of immune checkpoint molecules, compromised antigen presentation, and the presence of regulatory T cells (T_reg_).^7^

With the advent of immunotherapy, the optimal approach for treating advanced liver cancer has shifted towards combining immunotherapy with targeted or anti‐angiogenic treatment.^7^ According to the latest update of the Barcelona Clinic Liver Cancer (BCLC) algorithm,^8^ the combinations of atezolizumab and bevacizumab (Atezo‐Bev) and the combinations of durvalumab and tremelimuma (Durva‐treme) are the first‐choice first‐line treatment options, and if not feasible, sorafenib, lenvatinib, durvalumab, and palizumab are the secondary recommendations. Notably, the objective response rate (ORR) of immune checkpoint inhibitors (ICIs) in HCC is approximately 20%. This indicates that a significant proportion of individuals do not attain a response to this type of treatment.^9^ The ability to predict the efficacy of immunotherapy for liver cancer before treatment is of utmost importance for formulating the overall management program. Identifying reliable biological markers for selecting individuals with the highest probability of responding to immunotherapeutic drugs is of significant interest.^7^

Tumor‐infiltrating immune cells in the tumor immune microenvironment (TIME) may be related to the efficacy of immunotherapy for liver cancer,^10^, ^11^, ^12^, ^13^ which, however, can hardly be detected noninvasively, especially in patients receiving immunotherapy. The search for prediction indicators in peripheral blood has become a major research hotspot.^12^, ^13^, ^14^ It is assumed that changes in peripheral immunity also reflect alterrations in the TIME. Peripheral blood‐based parameters can provide some insights into the TIME and indicate the anticancer immune response of a host. The constitution of circulating white blood cells, including neutrophils, lymphocytes, and monocytes, may be linked to systemic inflammatory responses.^10^ For instance, a high neutrophil‐to‐lymphocyte ratio (NLR) is linked to poorer survival rates and a lower likelihood of response to immunotherapy for various advanced cancers.^15^, ^16^, ^17^, ^18^

This study aimed to reveal the associations of the three peripheral blood‐based indexes, including NLR, monocyte‐to‐lymphocyte ratio (MLR), and platelet‐to‐lymphocyte ratio (PLR), and their changes with the efficacy of immunotherapy for liver cancer. This study majorly focused on HCC, and cases with intrahepatic cholangiocarcinoma (ICC) were separately analyzed.

## 2 METHODS

### 2.1 Patients

Data analysis was conducted on patients with liver cancer who had not received any previous immunotherapy from four wards of the Department of Oncology in The First Affiliated Hospital of Anhui Medical University. Eligible individuals were those who received at least one dose of any kind of immunotherapy in the palliative setting from June 2019 to December 2020. Individuals were excluded if they had any other concurrent malignancy, recent infection, untreated HIV infection or (auto)immune diseases, antibiotic use, or corticosteroid administration within 7 days before the blood draw for NLR, MLR, and PLR assessments. These ratios were defined as the ratios of neutrophil, monocyte, and platelet count to lymphocyte count, respectively. In the main analyses, patients with intrahepatic cholangiocarcinoma (ICC) and those receiving adjuvant immunotherapy were also excluded.

In all patients, a routine examination of peripheral vein blood was performed shortly before the initiation of immunotherapy. The most recent differential blood cell counts both at baseline prior to immunotherapy initiation (at least 1 day before the first treatment) and before Cycle 2 of immunotherapy, along with the clinicopathological and treatment data, were extracted from the electronic medical records (EMR). This included information such as age, sex, body mass index (BMI), Eastern Cooperative Oncology Group (ECOG) Performance status (PS), smoking status, alcohol drinking status, histology, Barcelona clinic liver cancer (BCLC) staging, Child‐Pugh classification, history of Hepatitis B, history of cirrhosis, resection, chemotherapy, molecular targeted therapy, and immune checkpoint inhibitor (ICI) setting, number of metastasis sites, presence of extrahepatic metastasis, AFP level, and immunotherapy response data.^19^ Ratio data for other time points beyond Cycle 2 of immunotherapy were not included because only the first and second hematological evaluations were earlier than the imaging evaluation for response assessment, which was evaluated every 2 cycles of immunotherapy, and also because such ratio data were not available in most cases. ORR and disease‐control rate (DCR) were determined utilizing the Response Evaluation Criteria In Solid Tumors (RECIST; V.1.1).

This research received approval from the Ethics Committee of The First Affiliated Hospital of Anhui Medical University and adhered to the guidelines of the Declaration of Helsinki. Written informed consent was obtained from all participants.

### 2.2 Statistical analysis

Continuous variables were expressed as mean ± standard deviation; median (interquartile range) and compared between groups using the *t* or *U* test where appropriate. Categorical variables were shown as count (percentage [%]) and compared using *χ* ^2^ or Fisher's exact test where appropriate. We used the survcutpoint() function in the survminer package of R software to determine the optimal cutoffs for NLR, PLR, and MLR (BestNLR, BestPLR, and BestMLR), which were used to group NLR, MLR, and PLR into two subgroups.

OS was calculated from the start of immunotherapy to death due to any cause.^20^, ^21^, ^22^, ^23^ Individuals who remained alive at the data cutoff or last contact were censored. Progression‐free survival (PFS) was defined as the duration from the start of immunotherapy to disease progression, death, or last follow‐up, whichever came first. Censoring was applied to individuals who did not experience progression at the last disease assessment. The Kaplan–Meier method was applied to estimate the event‐time distribution, with survival compared using the log‐rank test. Multivariate Cox proportional hazard regression models were fitted to analyze the prognostic significances of the three ratios, with hazard ratios (HRs) and 95% confidence intervals (CIs) obtained. Statistical significance was predefined as two‐sided *P* < 0.050.

## 3 RESULTS

### 3.1 Characteristics of patients with HCC

A total of 167 patients with liver cancer were initially included. After excluding patients who had received previous immunotherapy (*n* = 9), those with any other concurrent malignancy (*n* = 12), those with untreated HIV infection or (auto)immune diseases (*n* = 5), those with recent infection (*n* = 3), those with antibiotic use (*n* = 4), those who had corticosteroid administration within 7 days before the blood draw for assessments of NLR, MLR, and PLR (*n* = 3), those with incomplete medical records (*n* = 1), those who were lost to follow‐up (*n* = 2), those receiving adjuvant immunotherapy (*n* = 9), and those with intrahepatic cholangiocarcinoma (ICC; *n* = 15), a total of 104 individuals with hepatocellular carcinoma (HCC) who received immunotherapy in the conventional setting met the eligibility criteria and were analyzed.

The baseline clinicopathological features of the analyzed individuals with HCC are shown in Table 1. The median age was 65, and 77.5% of the patients were male. 83.3% of the patients had an ECOG/PS score of 1,22.5% were current/former smokers, and 15.0% were current/former drinkers. 78.8% of the patients had a BCLC stage of C. 96.2% of the patients had a Child‐Pugh classification of B. In our hospital, most of the patients with early or middle stage operable HCC and good liver function were initially admitted to the Department of Hepatobiliary Surgery or the Department of Intervention. The patients with HCC who were admitted to the Department of Oncology mostly had an advanced cancer, and they were often complicated with cirrhosis, liver insufficiency, hypoproteinemia, ascites, and abnormal anticoagulant function, resulting in a poorer Child‐Pugh grade. In the real world, patients with a poorer Child‐Pugh grade (e.g., B) could still receive immunotherapy under meticulous care.^24^, ^25^, ^26^, ^27^, ^28^ 33.3% of the patients had a history of hepatitis B infection, 8.3% had positive HBV‐DNA, and 12.5% had cirrhosis. 46.2% of the patients underwent surgical resection, 8.7% received chemotherapy, with the regimens including gemcitabine, capecitabine, oxaliplatin, and paclitaxel, and 84.6% received molecularly targeted therapy. 93.3% of the patients used a programmed cell death‐1 (PD‐1) inhibitor (Table 1). 47.1% had two or more metastasis sites, and 63.5% had presence of extraheptic metastasis. Within the HCC patient cohort, the optimal cutoffs for baseline NLR, MLR, and PLR were 3.38, 0.28, and 227.18, respectively; in the ICC cohort, the optimal cutoffs for NLR, MLR, and PLR were 3.07, 0.27, and 193.68, respectively.

**TABLE 1:** Clinical and pathological characteristics of the included patients with hepatocellular carcinoma (HCC) receiving immunotherapy.

Characteristics	Overall cohort, *n* = 104
Age, >65 years	37 (35.6)
Sex, male	86 (82.7)
BMI	22.31 ± 3.63
ECOG performance status score	
0	6 (5.8)
1	87 (83.7)
2–4	11 (10.6)
Smoking status, current/former	24 (23.1)
Alcohol drinking status, current/former	15 (14.4)
BCLC	
0 or A	5 (4.8)
B	17 (16.3)
C	82 (78.8)
Child‐Pugh	
A	3 (2.9)
B	100 (96.2)
C	1 (1.0)
Cirrhosis, yes	14 (13.5)
History of hepatitis B, yes	39 (37.5)
HBV‐DNA postive, yes	8 (7.7)
Surgical treatment, yes	48 (46.2)
Chemotherapy, yes	9 (8.7)
Molecular targeted therapy, yes	88 (84.6)
Programmed cell death‐1 (PD‐1) inhibitor	97 (93.3)
NLR	3.50 ± 2.13
PLR	142.52 ± 72.03
MLR	0.39 ± 0.22
AFP (μg/L)	2411.17 ± 13371.73
Number of metastatic sites, ≥2	49 (47.1)
Presence of extrahepatic metastases	66 (63.5)

Abbreviations: BMI, body mass index; BCLC, Barcelona clinic liver cancer; ECOG, Eastern Cooperative Oncology Group; NLR, neutrophil‐to‐lymphocyte ratio; PLR, platelet‐to‐lymphocyte ratio; MLR, monocyte‐to‐lymphocyte ratio.

*Note*: Categorical variables are shown as count (percentage), continuous variables as mean ± standard deviation; median (interquartile range).

The median overall survival (mOS), median progression‐free survival (mPFS), and objective response rate (ORR) stratified by NLR, MLR, and PLR of the included patients with HCC are shown in Table 2.

**TABLE 2:** Median overall survival (mOS), median progression‐free survival (mPFS), and objective response rate (ORR) stratified by neutrophil‐to‐lymphocyte ratio (NLR), monocyte‐to‐lymphocyte ratio (MLR), and platelet‐to‐lymphocyte ratio (PLR) of the included patients with hepatocellular carcinoma.

Group	mOS (months)	mPFS (months)	ORR (%)
NLR≥3.38	7.70	3.93	7.69%
NLR <3.38	18.83	16.80	8.65%
MLR≥0.28	10.43	5.47	9.62%
MLR <0.28	25.5	NE	6.73%
PLR≥227.18	4.2	2.97	2.88%
PLR < 227.18	13.43	13.77	11.54%

Abbreviation: NE, not estimable.

### 3.2 NLR at baseline in the HCC cohort

**FIGURE 1:**
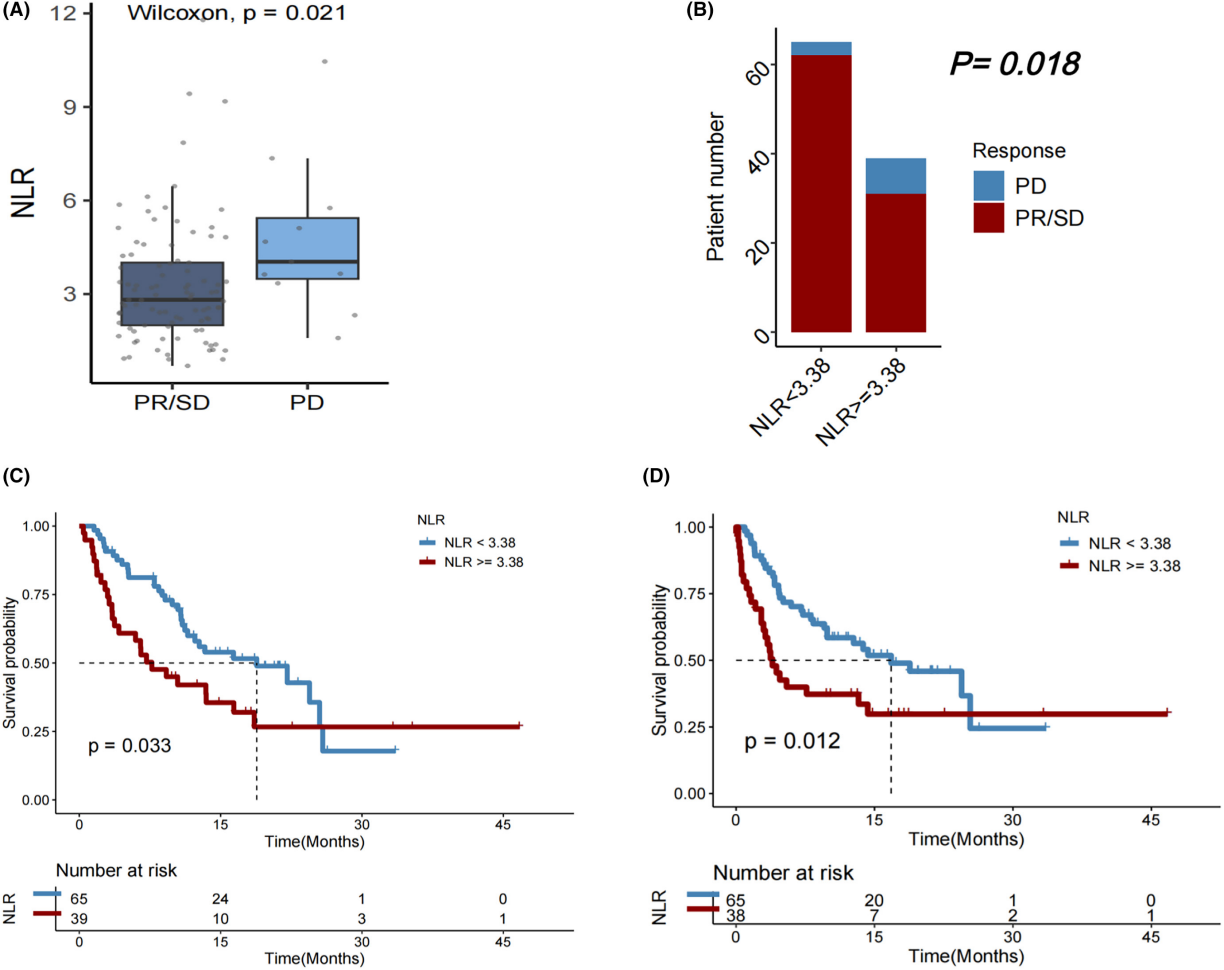
(A) Neutrophil‐to‐lymphocyte ratio (NLR) of individuals with HCC who experienced progressive disease (PD) versus partial response (PR)/stable disease (SD) as the best objective response to first immunotherapy. (B) Disease control rate, (C) overall survival (OS), and (D) progression‐free survival (PFS) in HCC patients with NLR < versus ≥3.38.

Among the 104 patients with HCC who received immunotherapy, those who experienced progressive disease (PD) after immunotherapy had higher median NLR compared to those with disease controlled, that is, a best objective response of partial response (PR) or stable disease (SD) (*p* = 0.021; Figure 1A). Accordingly, patients with NLR <3.38 had higher DCR (PR/SD rate) than those with NLR ≥3.38 (*p* = 0.018; Figure 1B). Both median OS (mOS) and median PFS (mPFS) were considerably longer in individuals with NLR <3.38 compared with those with NLR ≥3.38 group (*p* = 0.033 and 0.012, respectively; Figure 1C,D).

**FIGURE 2:**
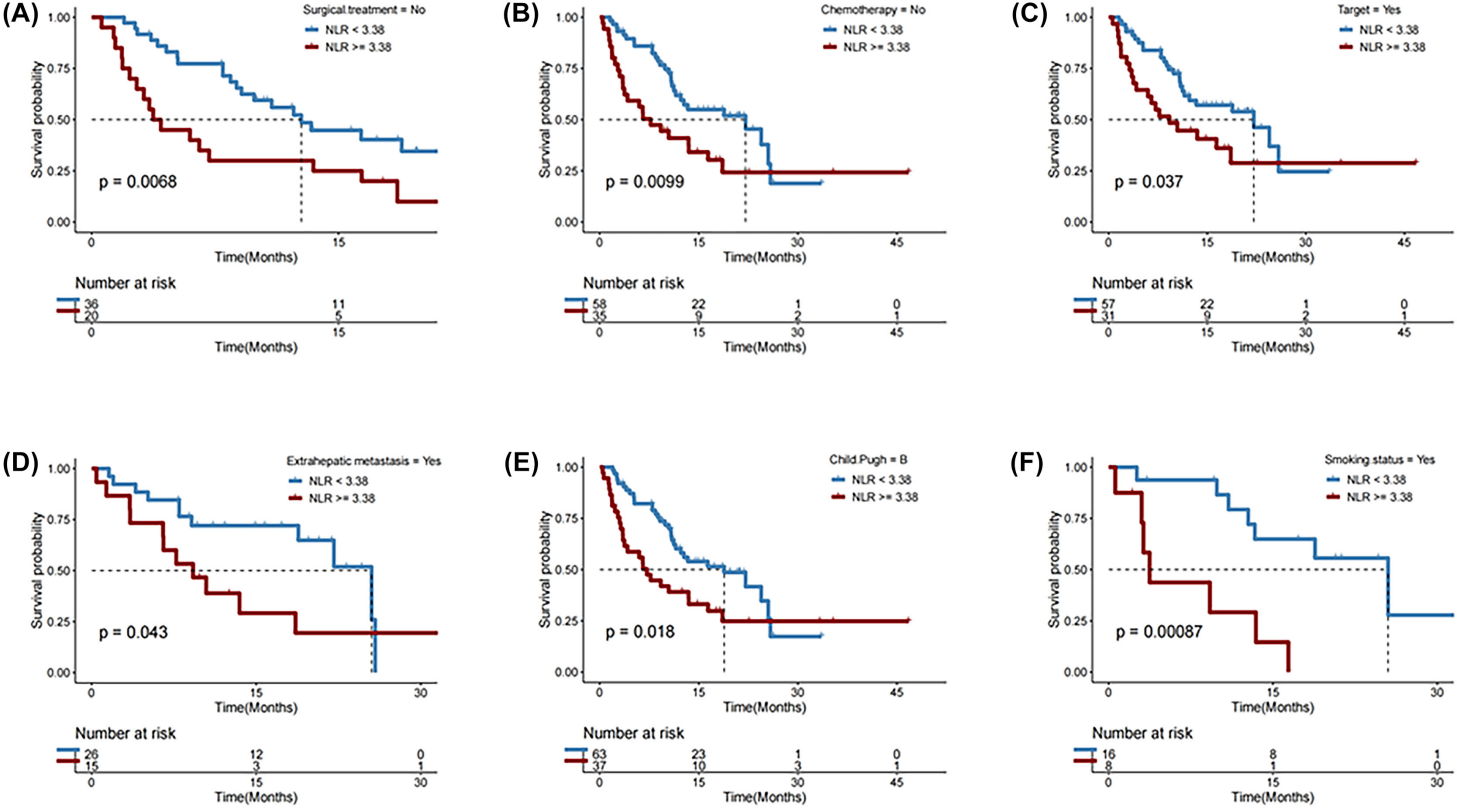
Overall survival (OS) in subgroups of HCC patients with neutrophil‐to‐lymphocyte ratio (NLR) < versus ≥3.38 (low vs. high).

In subgroups of patients who did not underwent surgery (*p* = 0.007; Figure 2A), those who did not receive chemotherapy (*p* = 0.010; Figure 2B), those who received targeted therapya (*p* = 0.037; Figure 2C), those with extraheptic metastasis (*p* = 0.043; Figure 2D), those with Child‐Pugh B liver function (*p* = 0.018; Figure 2E), and ever smokers (*p* = 0.001; Figure 2F), a lower NLR (<3.38) remained significantly associated with a longer OS after first immunotherapy.

After adjusting for age, sex, BMI, ECOG PS score, tobacco use, alcohol drinking, histology, BCLC staging, Child‐Pugh classification, history of Hepatitis B, resection, chemotherapy, molecular targeted therapy, and ICI setting, a higher NLR (≥3.38) was significantly and independently linked to a worse PFS (HR = 1.17, 95% CI = 1.03–1.33, *p* = 0.015).

Most baseline clinicopathological features, including age, sex, BMI, performance status, tobacco use, alcohol use, Child‐Pugh classification, history of cirrhosis and hepatitis B, HBV‐DNA status, surgical treatment, chemotherapy use, molecularly targeted therapy use, and the setting of ICI therapy, were balanced between the two subgroups with NLR < or ≥3.38, with no significant differences (Table S1). Patients with a lower NLR significantly had an earlier BCLC staging (*p* = 0.022), less frequent multiple metastatic sites (*p* = 0.027), and less frequent extraheptic metastasis (*p* = 0.011).

### 3.3 Association of early NLR change with efficacy after the first ICI immunotherapy

**FIGURE 3:**
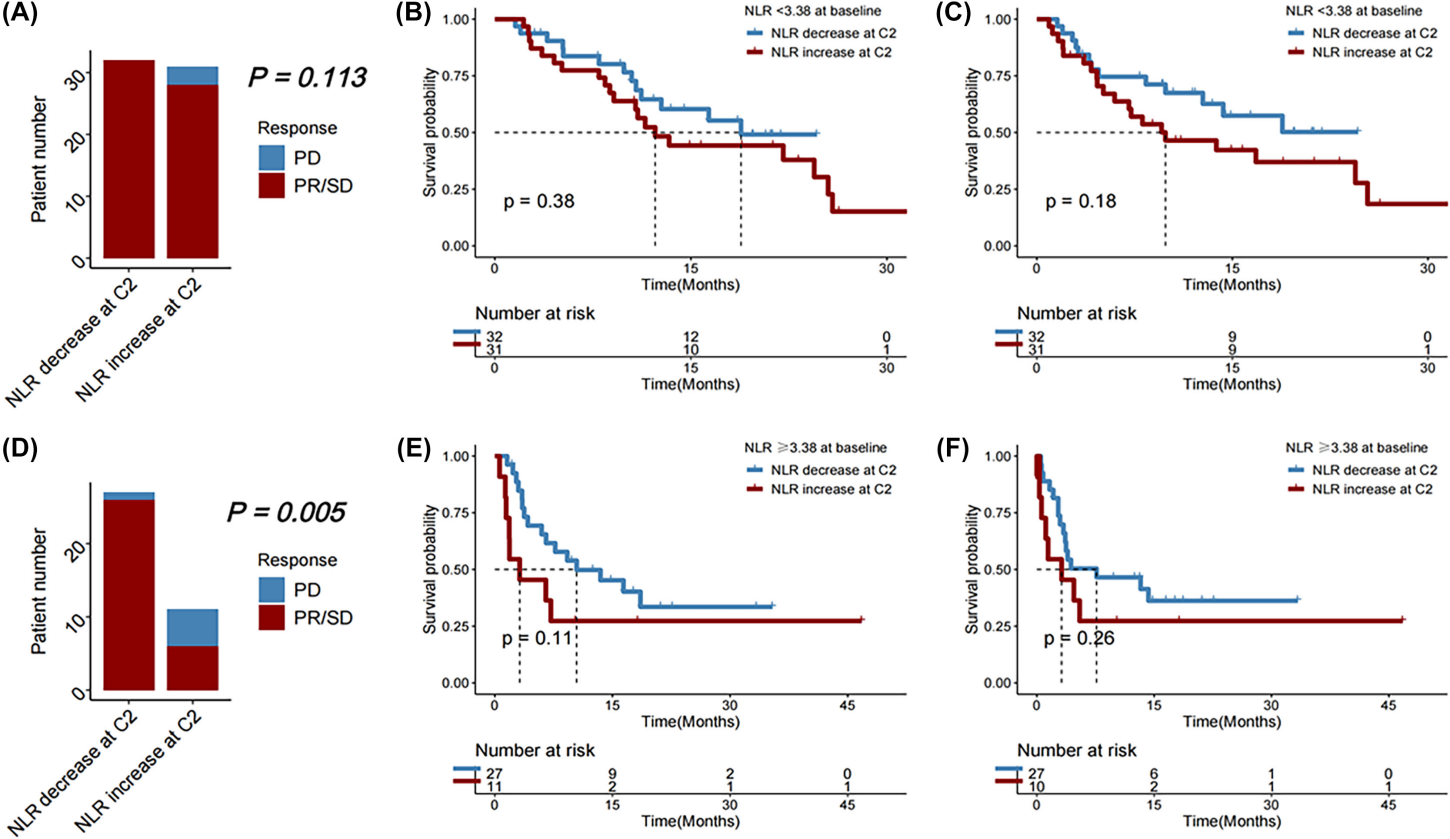
(A) Disease control rate (DCR), (B) overall survival (OS), and (C) progression‐free survival (PFS) in HCC patients receiving immunotherapy with baseline neutrophil‐to‐lymphocyte ratio (NLR) < 3.38, followed by a decrease versus an increase in NLR at Cycle 2 (C2) of immunotherapy. (D) DCR, (E) OS, and (F) PFS in HCC patients receiving immunotherapy with baseline NLR ≥3.38, followed by a decrease versus an increase in NLR at C2 of immunotherapy.

Next, the study examined whether the alteration in NLR during the second cycle of immunotherapy, compared to baseline, was linked to oncology and clinical outcomes (Figure 3). Among individuals with a favorable baseline NLR <3.38, the disease control rate did not significantly vary between those with a subsequent reduction in NLR at Cycle 2 (C2) of immunotherapy and those with an increase (*p* = 0.113); change in NLR was neither significantly associated with PFS (*p* = 0.180) nor with OS (*p* = 0.380). Among individuals who had an unfavorable baseline NLR (≥3.38) before initiating the first immunotherapy, those who experienced an increase in NLR at Cycle 2 exhibited significantly worse DCR with a significantly higher proportion of progressive disease (PD) (*p* = 0.005); but change in NLR was not significantly associated with OS (*p* = 0.110) or PFS (*p* = 0.260).

### 3.4 MLR at baseline in the HCC cohort

**FIGURE 4:**
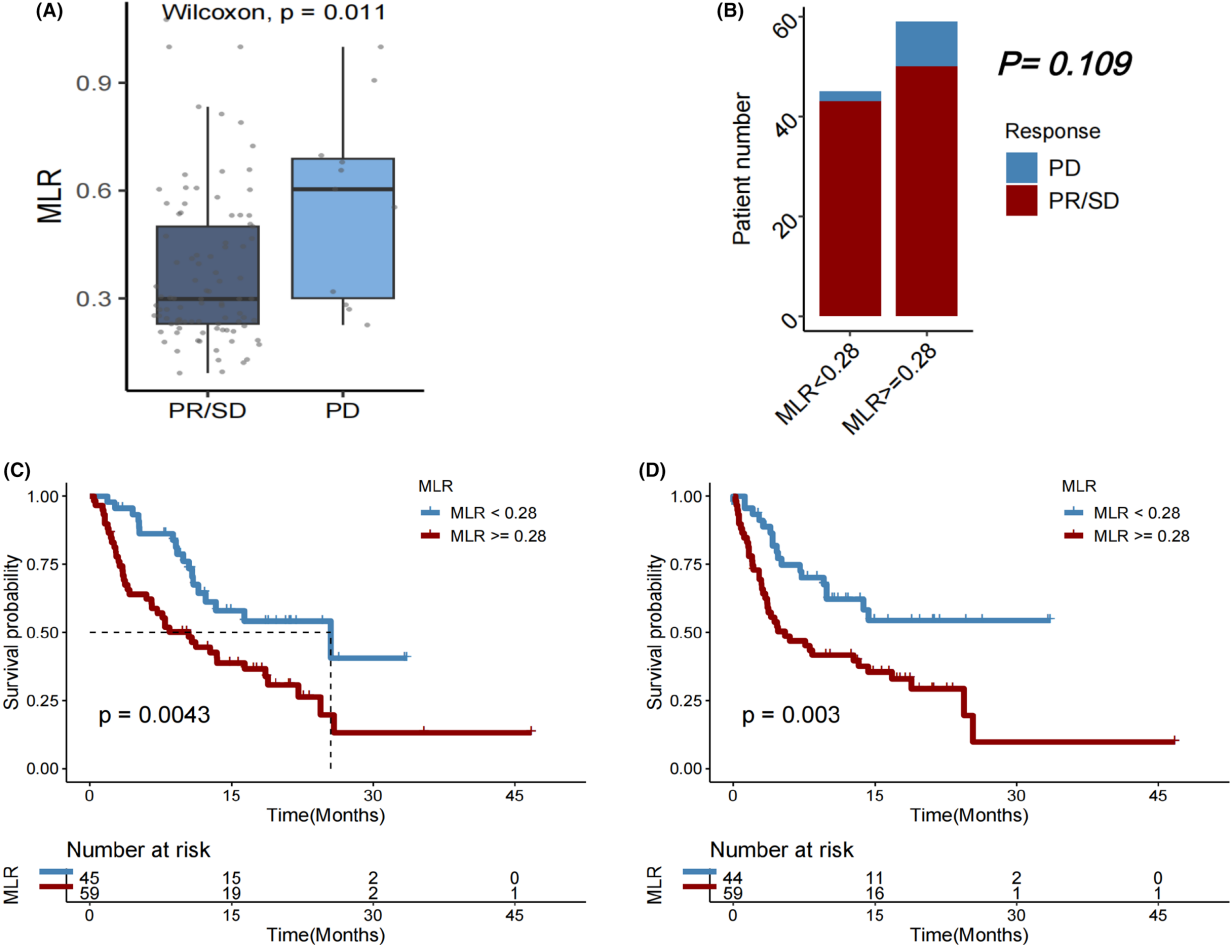
(A) Monocyte‐to‐lymphocyte ratio (MLR) in patients with HCC who experienced progressive disease (PD) versus partial response (PR)/stable disease (SD) as the best objective response to first immunotherapy. (B) Disease control rate (DCR), (C) overall survival (OS), and (D) progression‐free survival (PFS) in HCC patients with MLR < versus ≥0.28.

Patients who experienced a PD after immunotherapy significantly had a higher median MLR than those with a best objective PR or SD response, indicating disease control (*p* = 0.011, Figure 4A). However, DCR did not significantly vary between individuals with MLR <0.28 and those with MLR ≥0.28 (*p* = 0.109, Figure 4B). Both OS (*p* = 0.0043; Figure 4C) and PFS (*p* = 0.003; Figure 4D) after the first immunotherapy were significantly longer in individuals with baseline MLR <0.28 compared to those with MLR ≥0.28.

**FIGURE 5:**
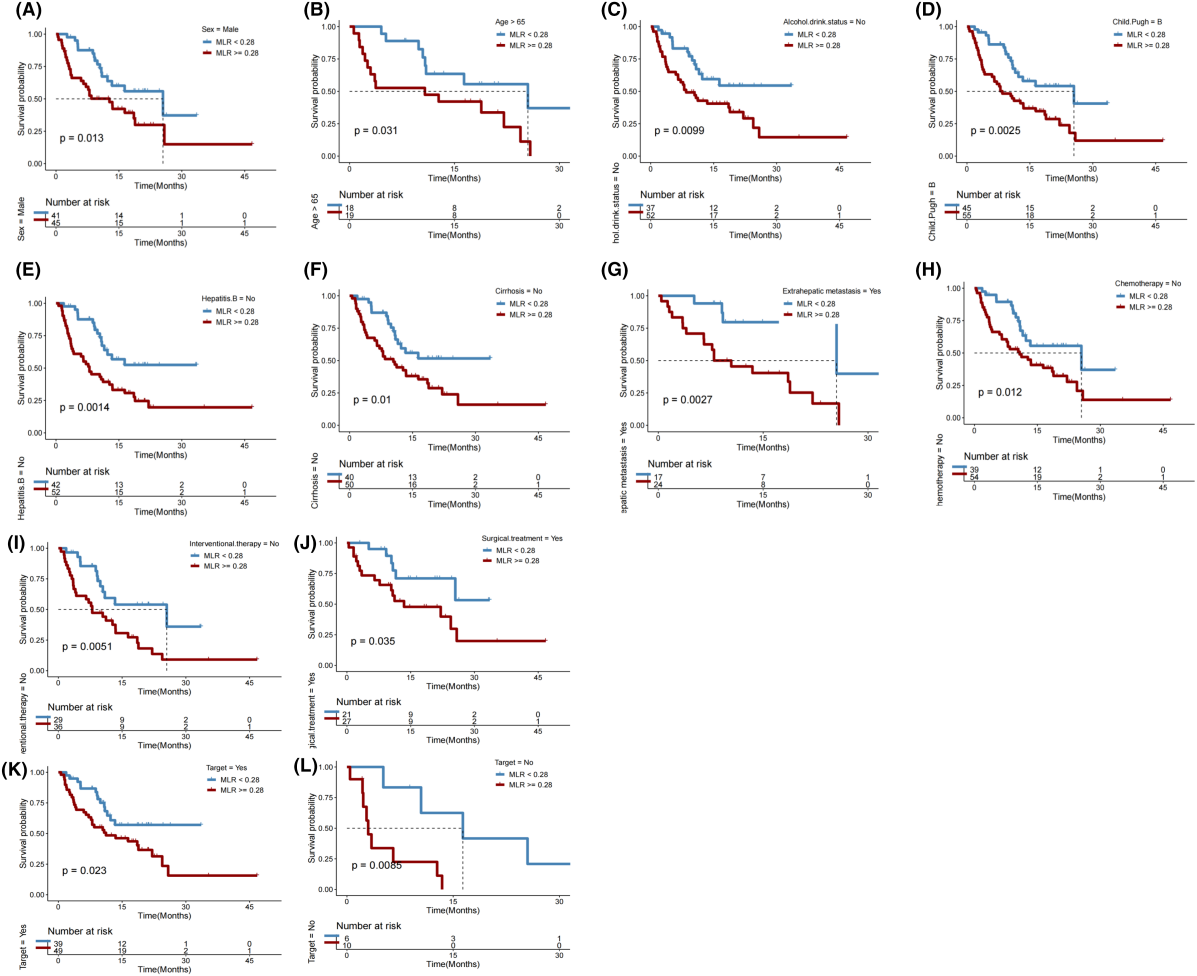
Overall survival (OS) in subgroups of HCC patients with monocyte‐to‐lymphocyte ratio (MLR) < versus ≥0.28.

In subgroups of male patients (*p* = 0.013; Figure 5A), those with age > 65 years (*p* = 0.031; Figure 5B), those who never drank alcohol (*p* = 0.010; Figure 5C), those with Child‐Pugh B liver function status (*p* = 0.003; Figure 5D), those who had no history of hepatitis B infection (*p* = 0.001; Figure 5E), those with no cirrhosis history (*p* = 0.010; Figure 5F), those with extrathepatic metastasis (*p* = 0.003; Figure 5G), those who did not receive chemotherapy (*p* = 0.012; Figure 5H), those who did not receive interventional therapy (*p* = 0.005; Figure 5I), those who underwent resection (*p* = 0.035; Figure 5J), those who received targeted therapy (*p* = 0.023; Figure 5K), and those who did not receive targeted therapy (*p* = 0.009; Figure 5L), a lower MLR (<0.28) remained significantly associated with a longer OS after first immunotherapy.

Most baseline clinicopathological features, including age, sex, BMI, tobacco use, alcohol use, Child‐Pugh classification, history of cirrhosis and hepatitis B, HBV‐DNA status, surgical treatment, chemotherapy use, molecularly targeted therapy use, and the setting of ICI therapy, were balanced between the two subgroups with MLR < or ≥0.28, with no significant differences (Table S2). Patients with a lower MLR significantly had a better performance status (*p* = 0.006) and an earlier BCLC stage (*p* = 0.007).

### 3.5 Early MLR change correlated with efficacy after the first ICI immunotherapy

**FIGURE 6:**
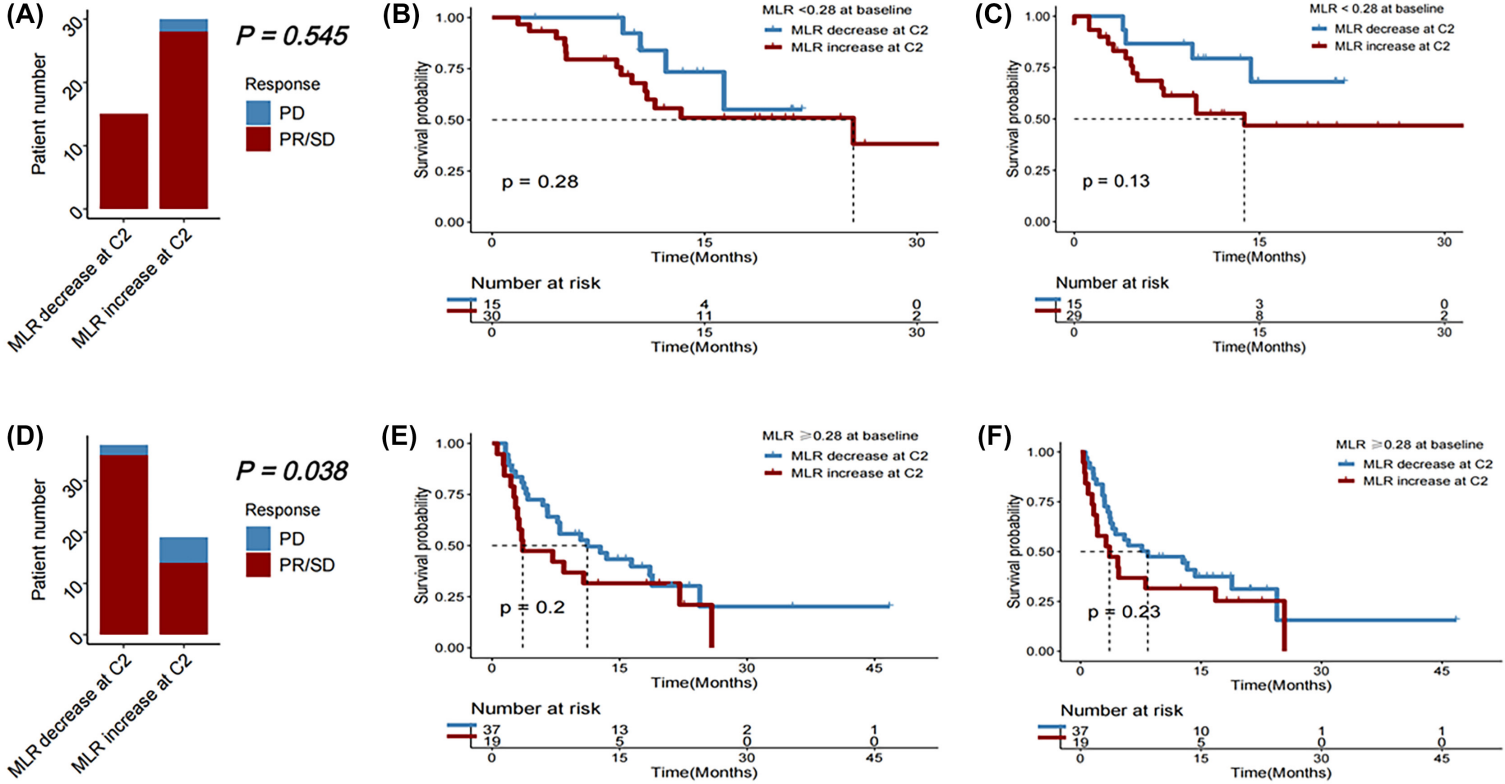
(A) Disease control rate, (B) overall survival (OS), and (C) progression‐free survival (PFS) in HCC patients receiving immunotherapy with baseline monocyte‐to‐lymphocyte ratio (MLR) < 0.28, followed by a decrease versus an increase in MLR at Cycle 2 (C2) of immunotherapy. (D) Disease control rate, (E) OS, and (F) PFS in HCC patients receiving immunotherapy with baseline MLR ≥0.28, followed by a decrease versus an increase in MLR at C2 of immunotherapy.

Change in MLR at C2 of immunotherapy compared to baseline did not significantly correlate with survival outcomes after the first immunotherapy (all *p* > 0.050). Among patients who initially had a favorable baseline MLR (<0.28), the DCR did not significantly vary between those with a subsequent reduction in MLR and those with an increase (*p* = 0.545). But in those with an unfavorable baseline MLR (≥0.28) prior to starting the first immunotherapy, a decrease in MLR at cycle 2 was significantly associated with better DCR (*p* = 0.038) (Figure 6).

#### 3.5.1 PLR at baseline in the HCC cohort

**FIGURE 7:**
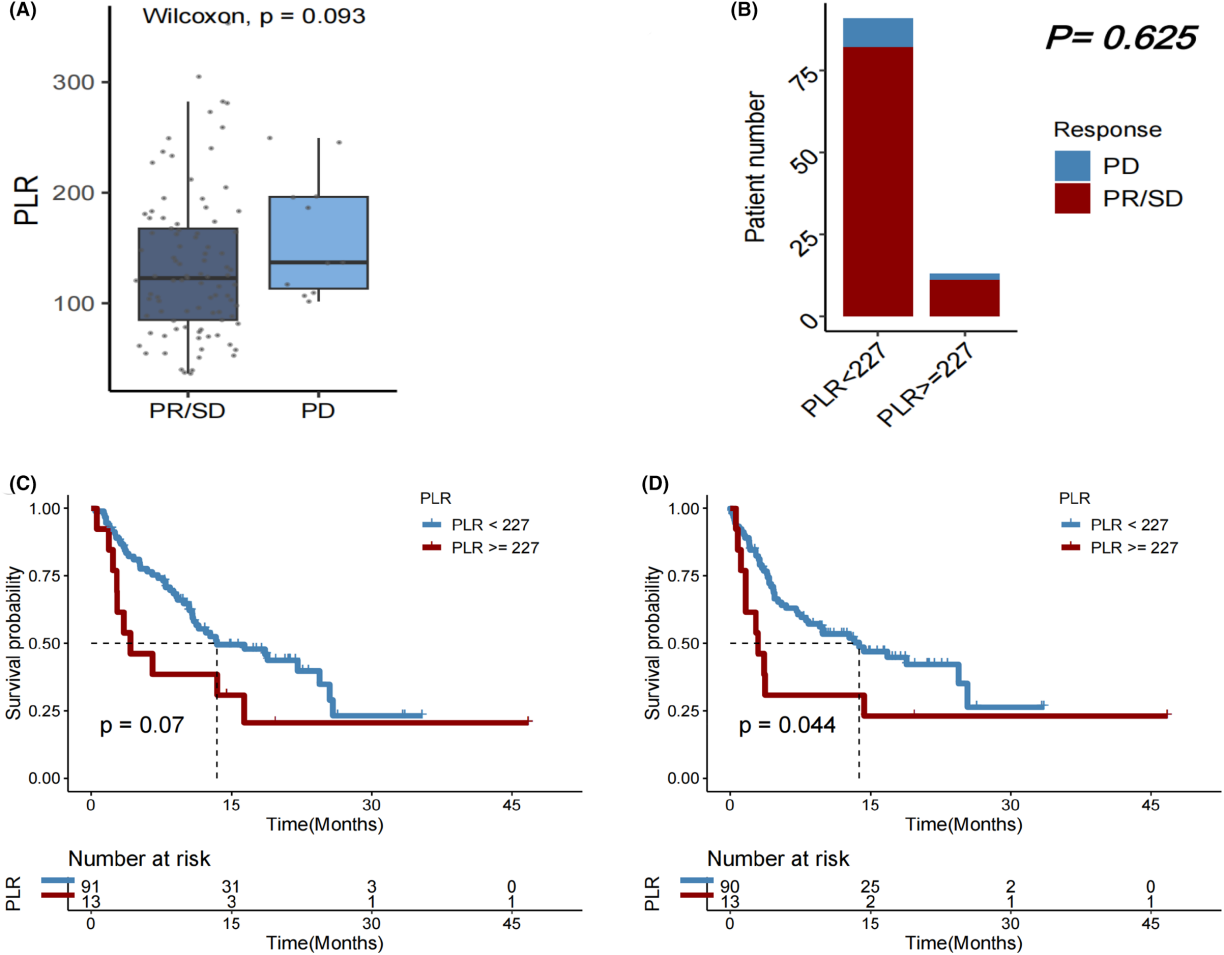
(A) Platelet‐to‐lymphocyte ratio (PLR) in patients with HCC who experienced progressive disease (PD) versus partial response (PR)/stable disease (SD) as the best objective response to first immunotherapy. (B) Disease control rate (DCR), (C) overall survival (OS), and (D) progression‐free survival (PFS) in HCC patients with PLR < versus ≥227.

**FIGURE 8:**
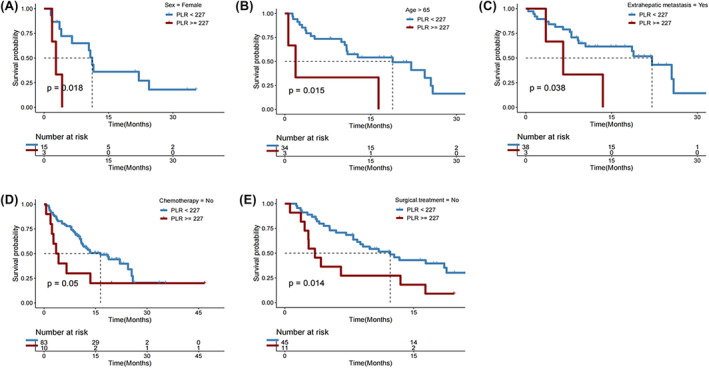
Overall survival (OS) in subgroups of HCC patients with platelet‐to‐lymphocyte ratio (PLR) < versus ≥227.

No considerable variations in PLR were observed between patients with PD and those with PR or SD after immunotherapy (*p* = 0.093; Figure 7A). Additionally, no significant variations in DCR (*p* = 0.625; Figure 7B) or OS (*p* = 0.070; Figure 7C) were observed between patients with PLR≥227.18 and those with PLR <227.18. But PFS was significantly longer in individuals with PLR <227.18 compared with those with PLR ≥227.18 (*p* = 0.005; Figures 7D).In subgroups of female patients (*p* = 0.018; Figure 8A), those with age > 65 years (*p* = 0.015; Figure 8B), those who had extrathepatic metastasis (*p* = 0.038; Figure 8C), those who did not receive chemotherapy (*p* = 0.050; Figure 8D), and those who underwent resection (*p* = 0.014; Figure 5E), a lower PLR (<227.18) remained significantly associated with a longer OS after first immunotherapy.

Most baseline clinicopathological features, including age, sex, BMI, performance status, tobacco use, alcohol use, BCLC staging, Child‐Pugh classification, history of cirrhosis and hepatitis B, HBV‐DNA status, chemotherapy use, molecularly targeted therapy use, and the setting of ICI therapy, were balanced between the two subgroups with PLR < or ≥ 227.18, with no significant differences (Table S3). Patients with a lower PLR significantly more often underwent surgery (*p* = 0.019).

### 3.6 Early PLR change correlated with efficacy after the first ICI immunotherapy

**FIGURE 9:**
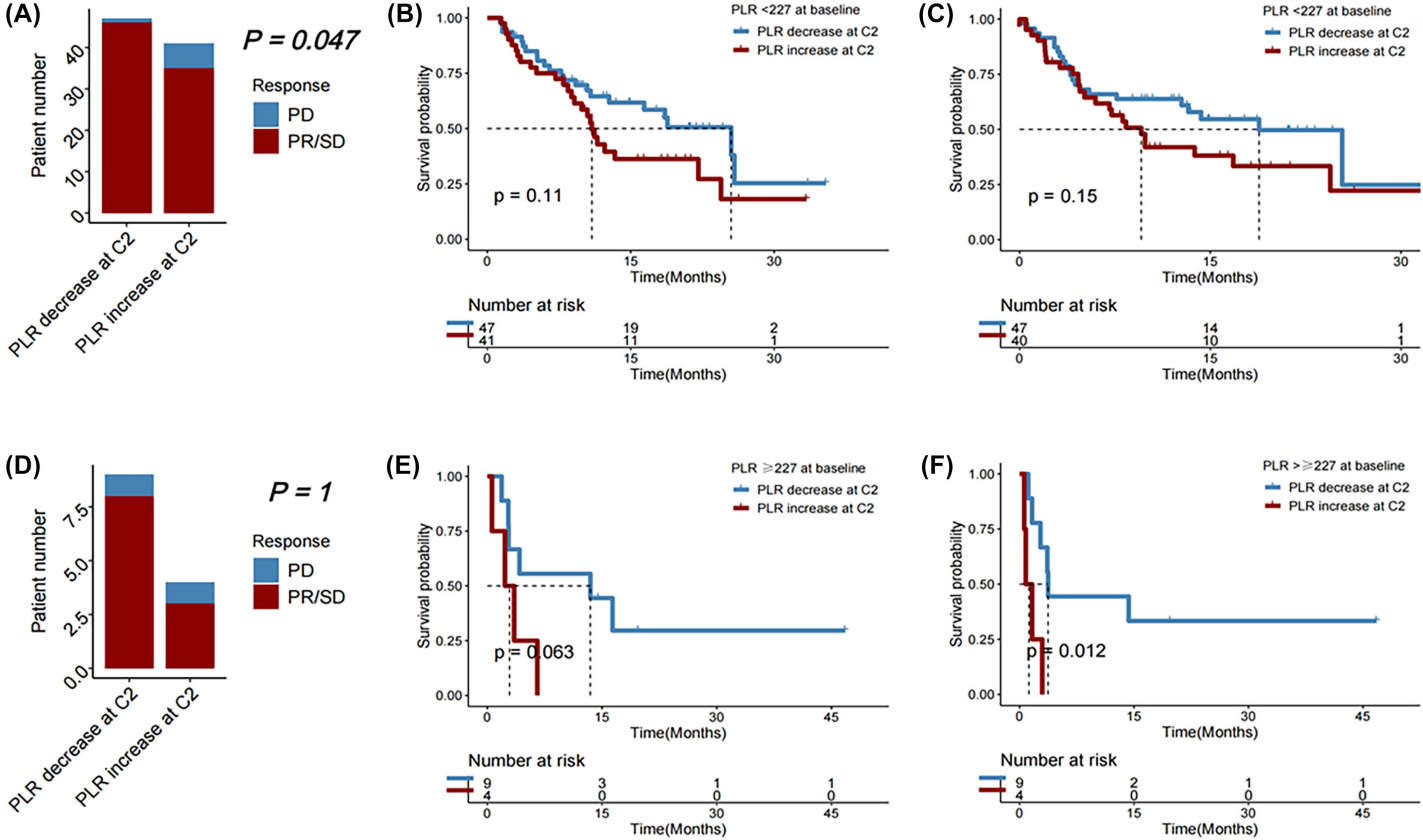
(A) Disease control rate, (B) overall survival (OS), and (C) progression‐free survival (PFS) in HCC patients receiving immunotherapy with baseline platelet‐to‐lymphocyte ratio (PLR) <227, followed by a decrease vs an increase in PLR at Cycle 2 (C2) of immunotherapy. (D) Disease control rate, (E) OS, and (F) PFS in HCC patients receiving immunotherapy with baseline PLR≥227, followed by a decrease versus an increase in PLR at C2 of immunotherapy.

Among patients with a favorable baseline PLR <227.18, a decrease in PLR was linked to a higher DCR (PR/SD rate) (*p* = 0.047), but subsequent change in PLR at cycle 2 of immunotherapy was not associated with OS or PFS (*p* = 0.110 and 0.150, respectively). Among patients with an unfavorable baseline PLR ≥227.18, a decrease in PLR at cycle 2 was significantly associated with longer PFS (*p* = 0.012), but change in PLR was not significantly associated with DCR (*p* = 1.000) or OS (*p* = 0.063) (Figure 9).

#### 3.6.1 NLR, MLR, and PLR in the ICC cohort

**FIGURE 10:**
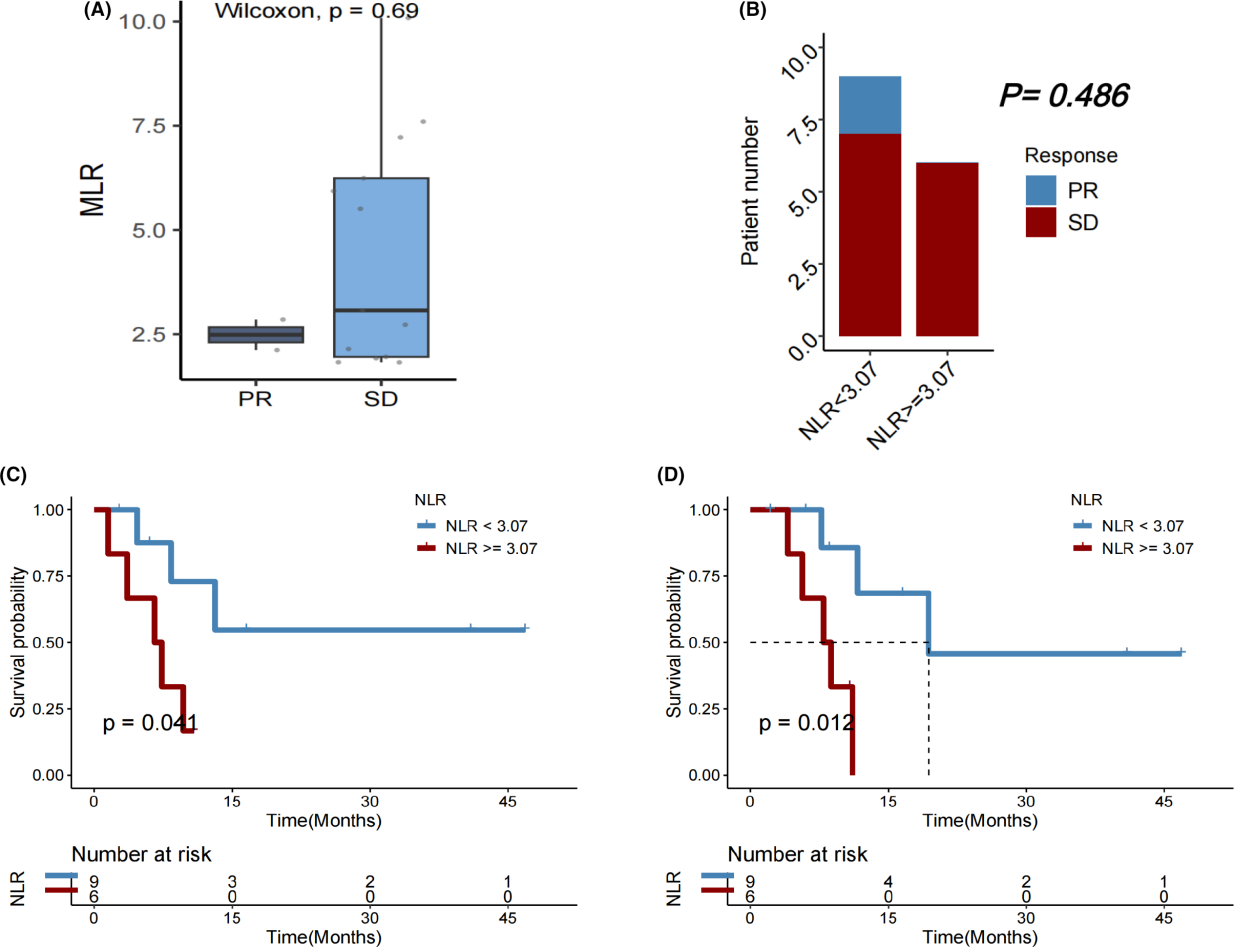
(A) Neutrophil‐to‐lymphocyte ratio (NLR) of individuals with ICC who experienced progressive disease (PD) versus partial response (PR)/stable disease (SD) as the best objective response to first immunotherapy. (B) Disease control rate, (C) overall survival (OS), and (D) progression‐free survival (PFS) in ICC patients with NLR < versus ≥3.07.

**FIGURE 11:**
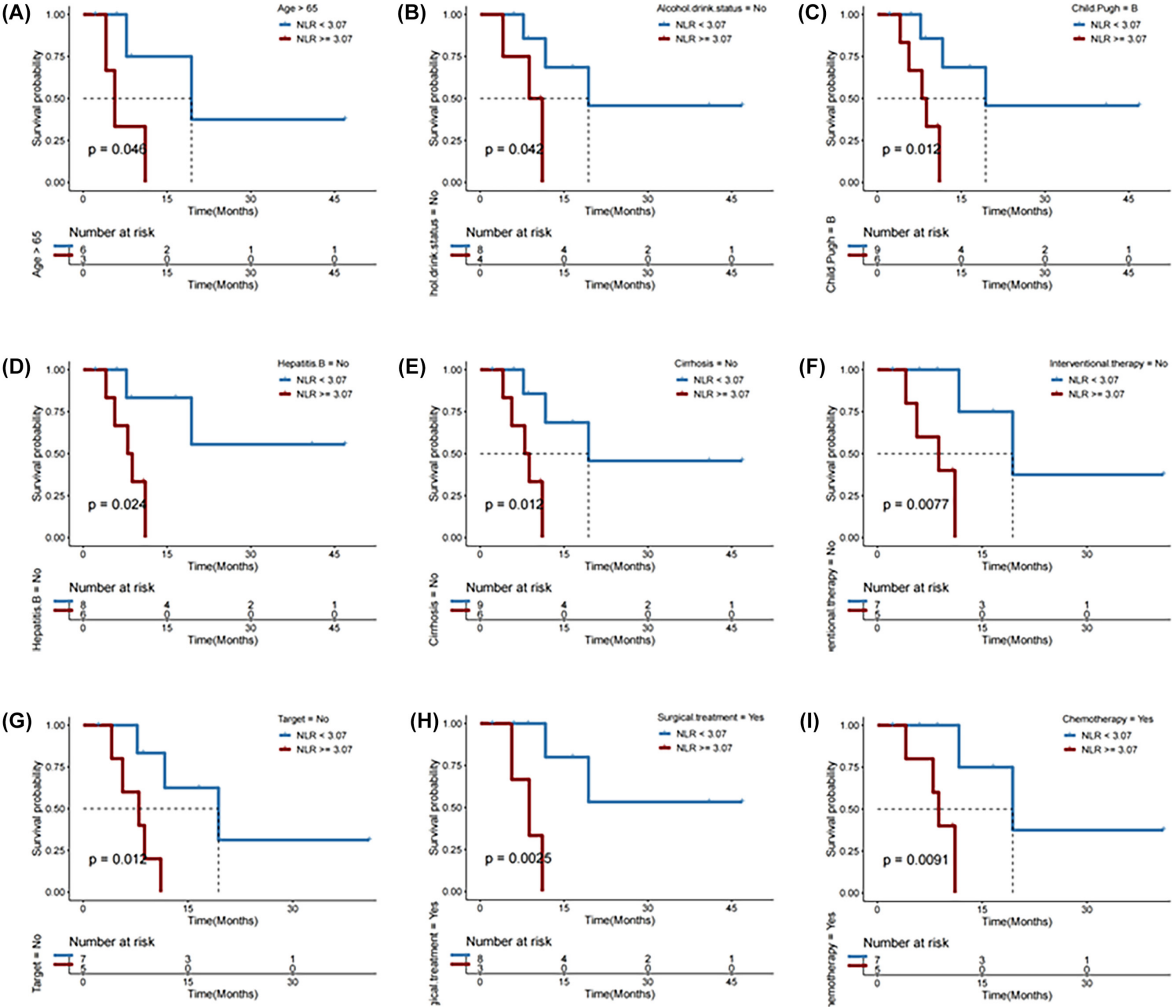
Overall survival (OS) in subgroups of ICC patients with neutrophil‐to‐lymphocyte ratio (NLR) < versus ≥3.07 (low vs. high).

**FIGURE 12:**
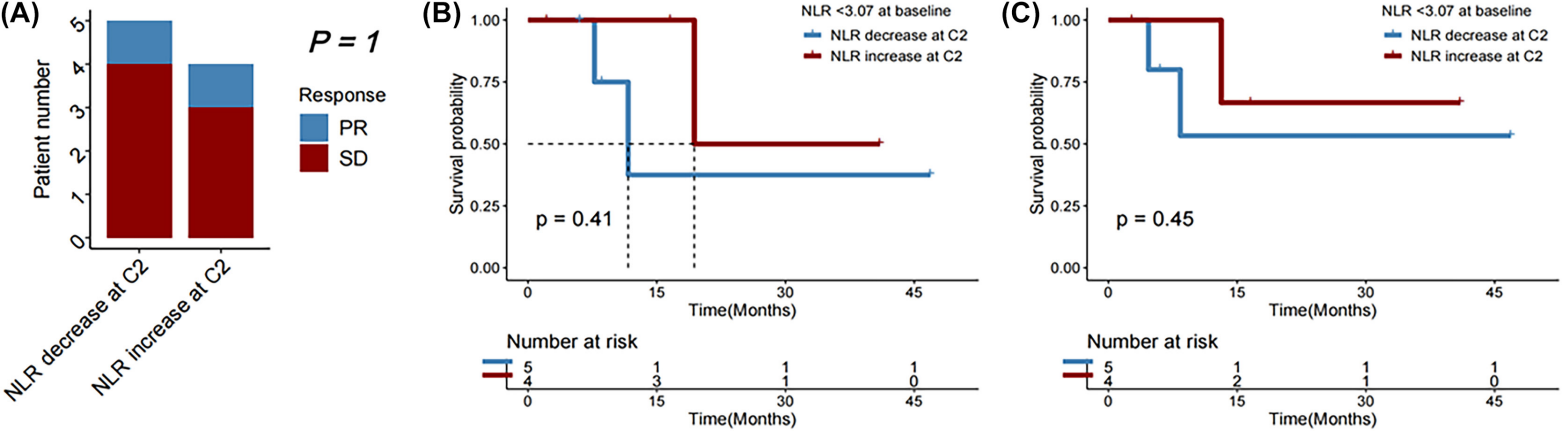
(A) Disease control rate (DCR), (B) overall survival (OS), and (C) progression‐free survival (PFS) in ICC patients receiving immunotherapy with baseline neutrophil‐to‐lymphocyte ratio (NLR) < 3.07, followed by a decrease vs an increase in NLR at Cycle 2 (C2) of immunotherapy.

**FIGURE 13:**
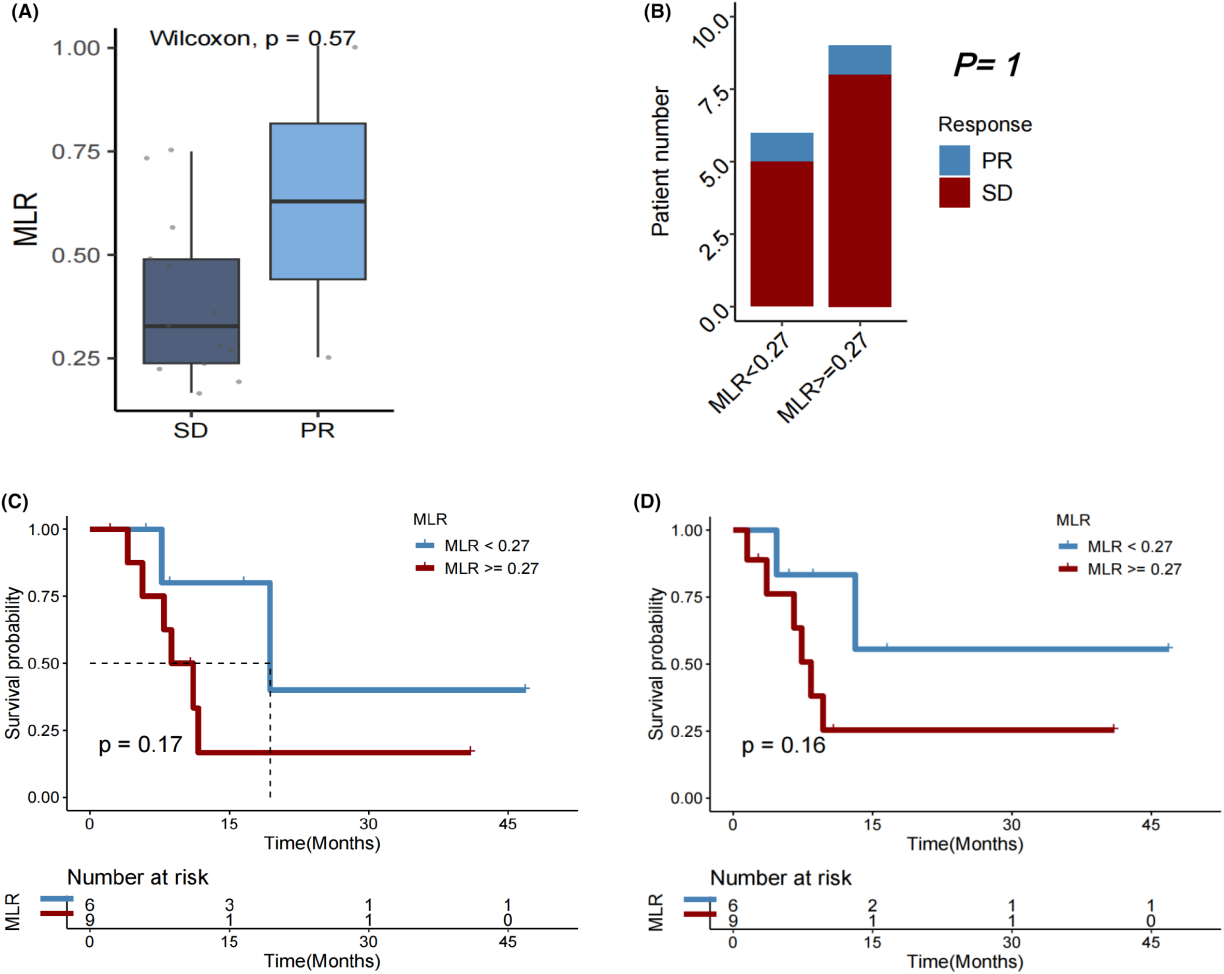
(A) Monocyte‐to‐lymphocyte ratio (MLR) in patients with ICC who experienced progressive disease (PD) versus partial response (PR)/stable disease (SD) as the best objective response to first immunotherapy. (B) Disease control rate (DCR), (C) overall survival (OS), and (D) progression‐free survival (PFS) in ICC patients with MLR < versus ≥0.27.

**FIGURE 14:**
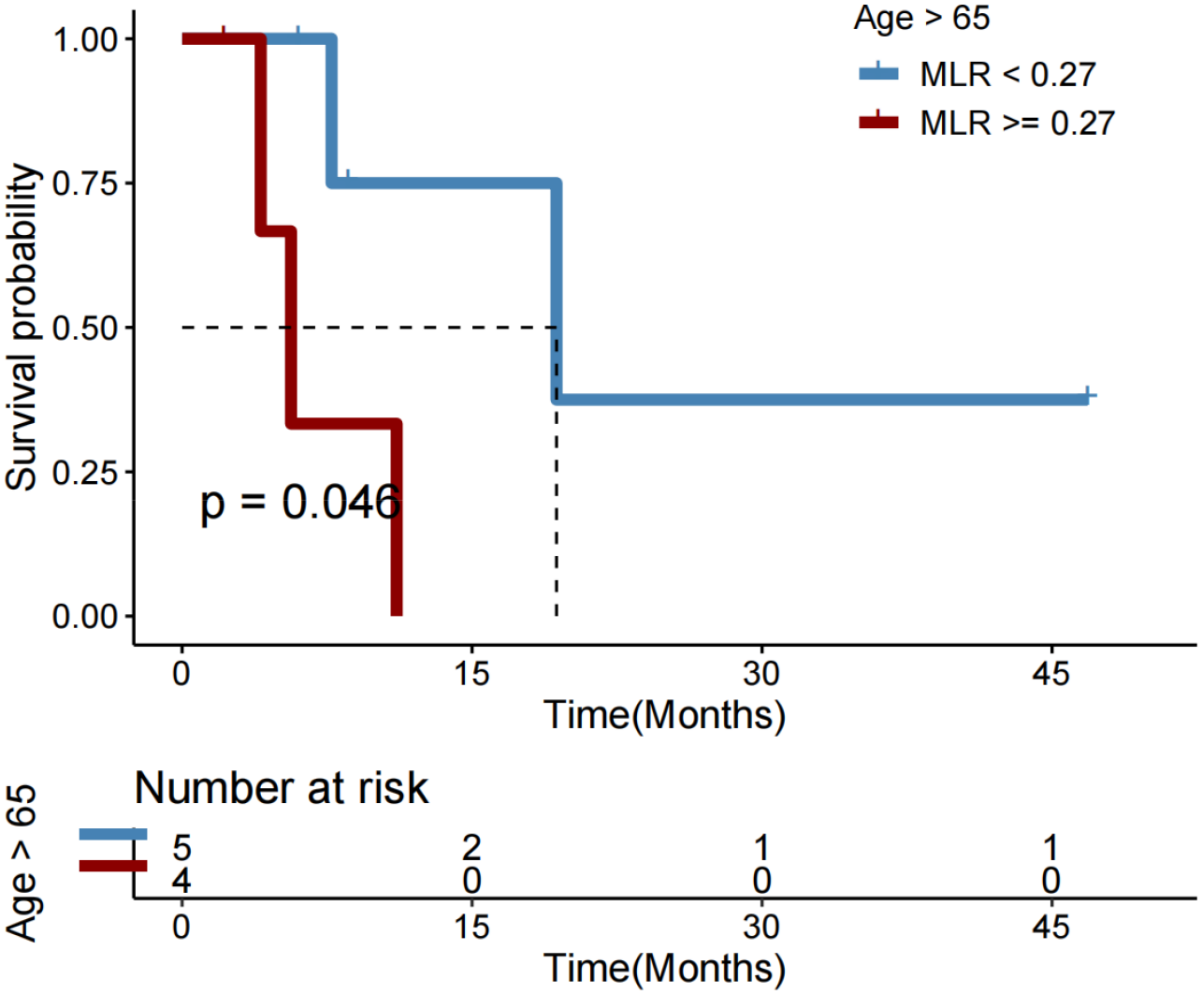
Overall survival (OS) in subgroups of ICC patients with monocyte‐to‐lymphocyte ratio (MLR) < versus ≥0.28.

**FIGURE 15:**
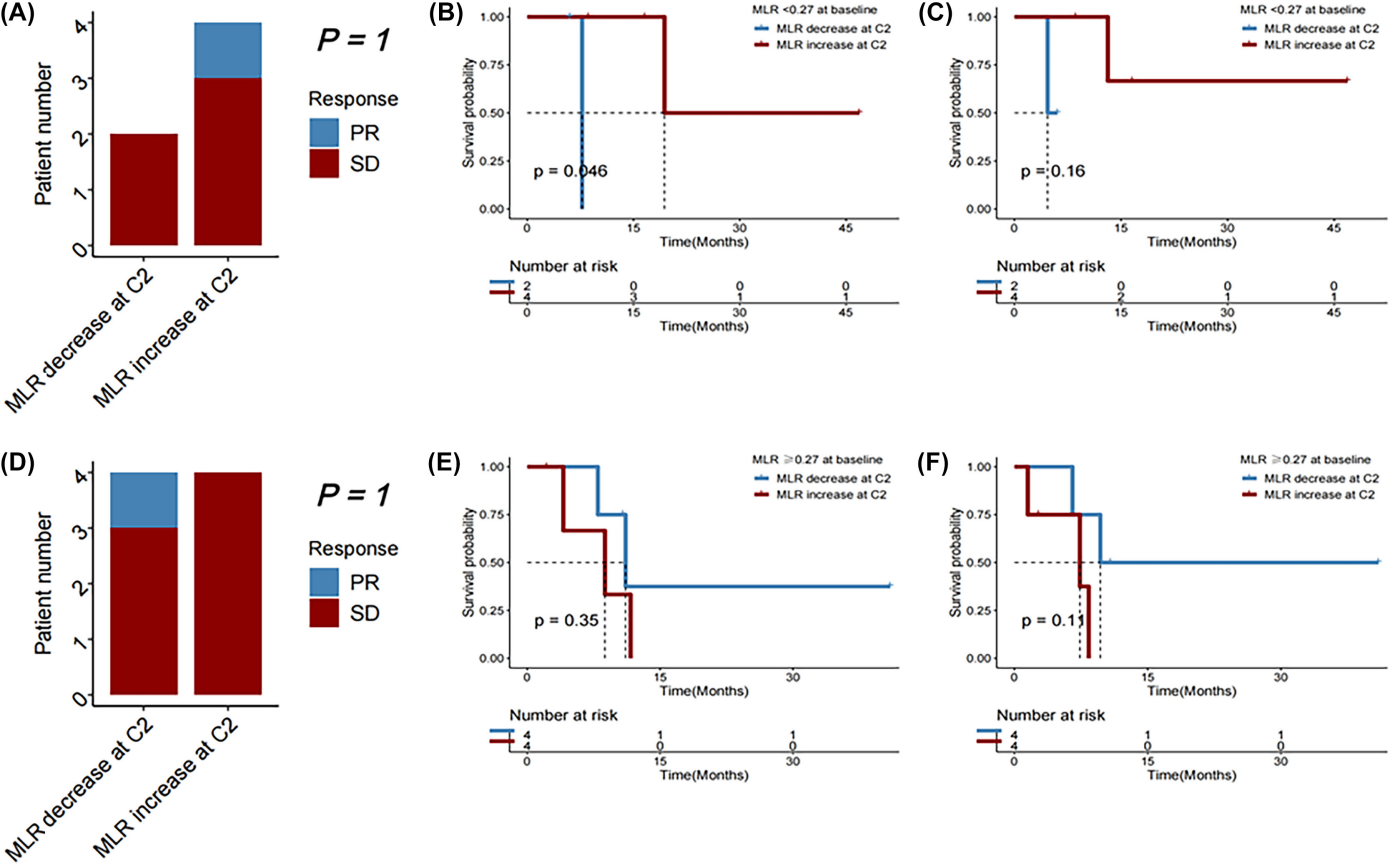
(A) Disease control rate, (B) overall survival (OS), and (C) progression‐free survival (PFS) in ICC patients receiving immunotherapy with baseline monocyte‐to‐lymphocyte ratio (MLR) < 0.27, followed by a decrease versus an increase in MLR at Cycle 2 (C2) of immunotherapy. (D) Disease control rate, (E) OS, and (F) PFS in ICC patients receiving immunotherapy with baseline MLR ≥0.27, followed by a decrease versus an increase in MLR at C2 of immunotherapy.

**FIGURE 16:**
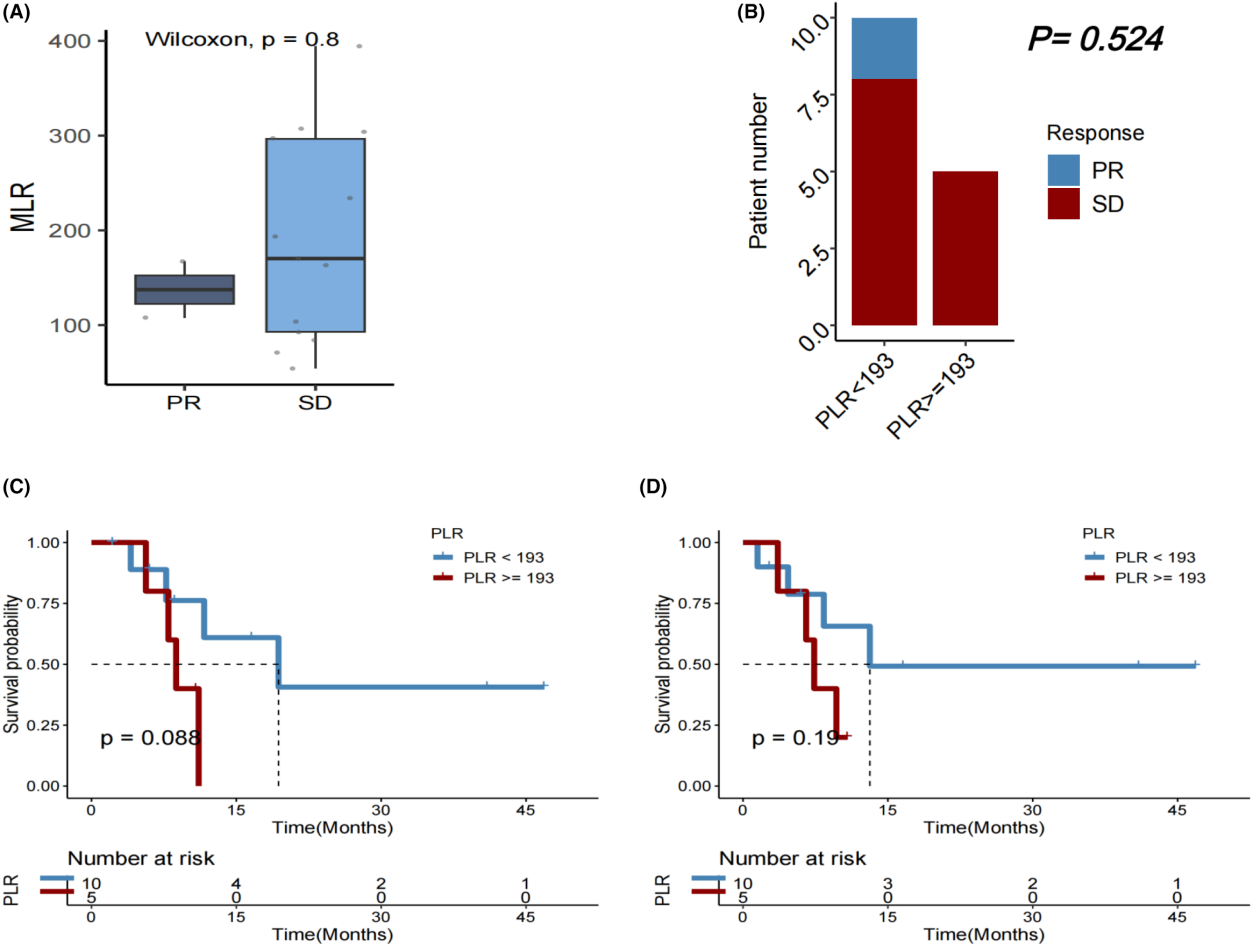
(A) Platelet‐to‐lymphocyte ratio (PLR) in patients with ICC who experienced progressive disease (PD) versus partial response (PR)/stable disease (SD) as the best objective response to first immunotherapy. (B) Disease control rate (DCR), (C) overall survival (OS), and (D) progression‐free survival (PFS) in ICC patients with PLR < versus ≥193.

**FIGURE 17:**
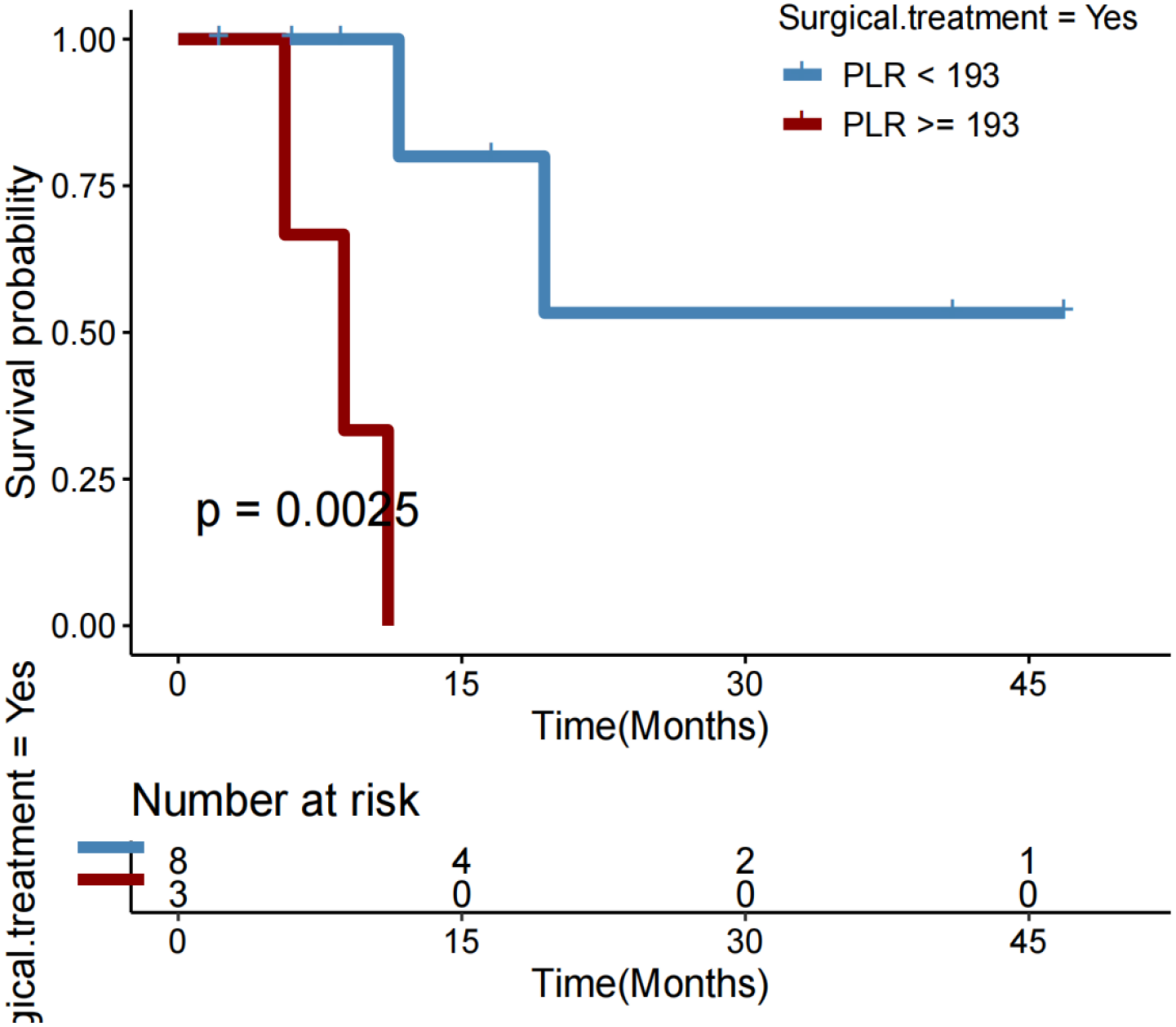
Overall survival (OS) in subgroups of HCC patients with platelet‐to‐lymphocyte ratio (PLR) < versus ≥193.

**FIGURE 18:**
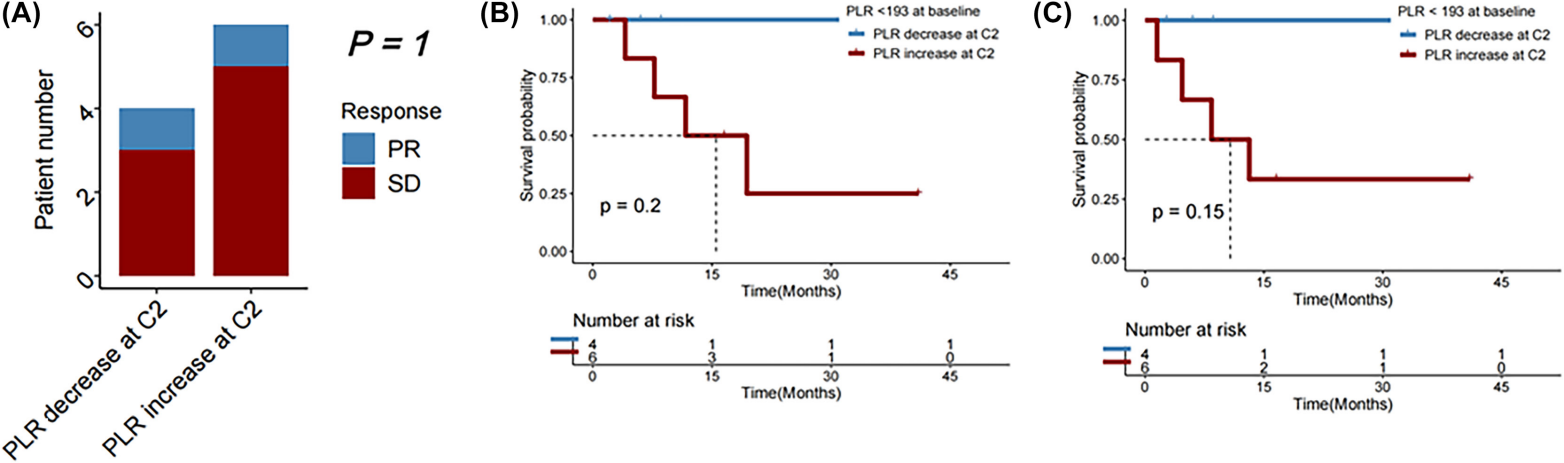
(A) Disease control rate, (B) overall survival (OS), and (C) progression‐free survival (PFS) in ICC patients receiving immunotherapy with baseline platelet‐to‐lymphocyte ratio (PLR) <193, followed by a decrease versus an increase in PLR at Cycle 2 (C2) of immunotherapy.

Among the 15 patients with ICC who received immunotherapy, both median OS (mOS) and median PFS (mPFS) were significantly longer in individuals with NLR <3.07 compared with those with NLR ≥3.07 (*p* = 0.041 and 0.012, respectively; Figure 10). In subgroups of patients with age > 65, those who never drank alcohol, those with Child‐Pugh B liver function, those with no hepatitis B infection, those with no cirhosis history, those who never received interventional therapy, those who did not receive targeted therapy, those who received surgical treatment, and those who received chemotherapy, a lower NLR (<3.07) remained significantly associated with a longer OS after first immunotherapy (all *p* < 0.050; Figure 11). Change in NLR, and baseline values of and changes in MLR and PLR were mostly not significantly associated with disease control or survival (Figures 12, 13, 14, 15, 16, 17, 18), likely due to the small case number analyzed.

## 4 DISCUSSION

This study showed that the level of peripheral blood immunomarkers, NLR, and MLR, were lower in patients with primary HCC who received immunotherapy with disease subsequently controlled (the best response of PR or SD) compared to those with PD after immunotherapy. Additionally, the study observed that with the cutoffs of 3.38 and 0.28 for NLR and MLR, respectively, lower NLR and MLR predicted improved oncology and clinical outcomes (higher DCR and longer OS and PFS) in overall patients and multiple subgroups. Furthermore, lower PLR (<227.18) was significantly associated with longer PFS. In the subgroup with an unfavorable baseline NLR ≥3.38 or MLR ≥0.28, or a favorable baseline PLR <227.18, a decrease in NLR, MLR, or PLR at Cycle 2 of immunotherapy was significantly associated with a higher DCR. In cases with ICC, a high baseline NLR (≥3.07) was correlated with worse PFS and OS.

There have been some previous reports on the significances of NLR and PLR in patients with HCC receiving immunotherapy. Muhammed et al.^29^ investigated the association of baseline NLR and PLR with prognosis of patients with HCC receiving immunotherapy majorly from Europe and the United States. Wu et al.^30^ explored the association of baseline NLR and PLR with prognosis in patients with HCC receiving specifically atezolizumab (a PD‐L1 inhibitor) plus bevacizumab, and the patients were also majorly from Europe and the United States. Our study provides the following advances^1^: all the investigated patients were Chinese^2^; the association of MLR with prognosis were also investigated^3^; we further explored the prognostic significances of changes of NLR, MLR, and PLR at Cycle 2 of immunotherapy compared to baseline^4^; the prognostic significances of NLR, MLR, and PLR at both baseline and Cycle 2 of immunotherapy were also explored in a subgroup of patients with intrahepatic cholangiocarcinoma (ICC).

In the study by Muhammed et al.^29^ using different types of immunotherapy for HCC, patients with PLR ≥300 had shorter OS (6.4 vs. 16.5 months, *p* < 0.001) and PFS (1.8 vs. 3.7 months, *p* = 0.001). In the study by Wu et al.^30^ focusing specifically at Atezolizumab‐Bevacizumab, PLR ≥300 was also significantly associated with decreased OS (9.4 vs. 15.7 months, *p* = 0.007) and PFS (3.5 vs. 7.1 months, *p* = 0.040) compared to PLR <300. In this study focusing on Chinese patients treated with immunotherapy for HCC, cases with PLR ≥227 also significantly had shorter PFS compared with their counterparts (3.0 vs. 13.8 months, *p* = 0.044), but PLR was not significantly associated with OS (*p* = 0.070). The difference between our study and the previous ones^29^, ^30^ in the prognostic role of PLR may be partly explained by the differences in ethnicity and sample size.

Presently, the recommended first‐line treatment for advanced liver cancer is a combination of immunotherapy and targeted therapy, which, however, is effective in less than half of the patients receiving the combination therapy. There remain many controversies concerning the merits of immunotherapy‐based monotherapy and combined therapy. It was found that patients with lower NLR, MLR, or PLR had better outcomes after immunotherapy, which may help to better select patients for immunotherapy, thus avoiding the potential unnecessary toxicity of immunotherapy to a certain extent. In prior studies, it was revealed that suppressed lymphocyte‐mediated immunity could reduce response to ICI in hepatocellular carcinoma (HCC).^31^

Similar findings supporting the beneficial role of lymphocytes and the tumor‐promoting role of neutrophils have been reported in cancers. Previous reports demonstrated that elevated neutrophil counts in both the peripheral blood and the tumor microenvironment strongly predicted poorer prognosis in individuals with cancer and that neutrophils could promote angiogenesis in cancer.^3^ In individuals with resected nonmetastatic Siewert type II/III adenocarcinoma of esophagogastric junction^12^ or gastric cancer,^13^ it was found that preoperative peripheral blood NLR and MLR with the exception of PLR negatively predicted clinical outcomes. Additionally, it was found that a higher posttreatment NLR was associated with poorer outcomes in individuals with advanced cancer who received PD‐1 antibody‐based immunotherapy.^32^ This research also discovered that a lower NLR was linked to more favorable clinicopathologic features, including higher BMI and better performance status. Lee et al^10^ demonstrated that a low lymphocyte‐to‐monocyte ratio (LMR) was significantly associated with poor OS and DFS among patients with breast cancer. Monocytes represent a significant immunosuppressive component within the leukocyte infiltrate in the tumor stroma.^33^ In various cancer patient populations, higher levels of monocytes within the tumor are typically linked to advanced clinical stages and unfavorable outcomes. Therefore, targeting monocytes presents a crucial therapeutic approach for tumor immunotherapy.^34^ Onagi et al.^35^ suggested that the combined assessment of PLR with tumor‐infiltrating lymphocyte (TIL) might enable more accurate prediction of outcomes of patients with triple‐negative breast cancer.

Notably, it was further discovered that NLR and MLR were more accurate predictors of survival in certain patient subgroups. For instance, in patients with extrahepatic metastasis and those with Child‐Pugh B liver function, both lower NLR and MLR were associated with higher OS. Notably, extrahepatic metastasis andworse liver functionmay interfere with immunotherapy efficacy and immunomarker levels. This could help in more precisely screening patients with liver cancer who might benefit from immunotherapy.

This study observed that in HCC patients with baseline NLR ≥3.38, MLR ≥0.28, or PLR <227.18 prior to first immunotherapy, an decrease in NLR, MLR, or PLR at Cycle 2 of immunotherapycompared to baseline was significantly associated with a higher DCR. These findings may aid in identifying individuals with liver cancer who are at a greater risk for disease progression and/or worse survival prior to the formulation of the next immunotherapy plan. However, Alessi et al.^36^ discovered that an early increase in NLR at Cycle 2 of pembrolizumab compared to baseline was linked to worse clinical outcomes in individuals with non‐small cell lung cancer and with a baseline NLR ≥2.6. Through single‐cell RNA sequencing (scRNA‐seq), a general increase in circulating neutrophil count in peripheral blood and a phenotype transition of neutrophils were previously observed after immune checkpoint inhibitor (ICI) immunotherapy in advanced gastric cancer.^32^ The discrepancies in the prognostic and predictive roles of changes in NLR between different courses of immunotherapy across different cancers may involve complex crosstalk mediated by various chemokines and intracellular signaling between immune cells and tumor cells. Investigating the underlying mechanisms underlying these discrepancies holds significant research value.

NLR and MLR are easily available prognostic and predictive biomarkers in the clinical setting. Assessment of NLR and MLR in the peripheral blood of patients with liver cancer may have implications for immunotherapy decision‐making. It may aid in guiding the design of immunotherapy‐based clinical trials, and direct future research in the relevant area.

This research has certain limitations due to its retrospective nature. The sample size of the study was limited, and the participation site was restricted to a single center, implying that the findings should be validated in larger independent cohorts of liver cancer patients, preferably in a prospective multicenter setting. It is hard to find many original images to remeasure tumor diameter, which was missing in most cases, and which is less significant compared to other clinicopathologic parameters in patients with advanced or metastatic HCC receiving immunotherapy. Additionally, the unavailability of original pathological specimens from patients with advanced liver cancer hindered further investigation through histological immunostaining or detection of tumor mutation burden (TMB) or tumor‐infiltrating immune cells. Further exploration is also needed to understand the relevant fundamental principles pertaining to the tumor immune microenvironment (TIME), and the correlation between peripheral immunity and the TIME. The cutoff of NLR (NLR = 5) as used in the previous publications,^29^, ^30^ which majorly analyzed patients from Europe and the United States, did not significantly predict overall survival or progression‐free survival in the Chinese patients with HCC receiving immunotherapy in this study (Figure S1). This may partly be explained by the difference in the ethnicity of the study population.

## 5 CONCLUSIONS

Among patients with primary HCC who received immunotherapy, lower baseline NLR, and MLR in overall patients were significantly associated with more favorable oncology and clinical outcomes (both OS and PFS) after immunotherapy, and lower PLR was significantly associated with longer PFS. The findings of this research may offer useful hints for a more efficient selection of patients with liver cancer who may benefit more from immunotherapy.

## AUTHOR CONTRIBUTIONS

**Sha Liu:** Conceptualization (equal); data curation (equal); formal analysis (equal); investigation (equal); methodology (equal); resources (equal); validation (equal); visualization (equal); writing – original draft (equal); writing – review and editing (equal). **Wentao Xu:** Data curation (equal); formal analysis (equal); investigation (equal); methodology (equal); software (equal); validation (equal); visualization (equal); writing – review and editing (equal). **Hang Shu:** Data curation (equal); formal analysis (equal); investigation (equal); methodology (equal); software (equal); validation (equal); visualization (equal); writing – review and editing (equal). **Ying Dai:** Investigation (equal); validation (equal); writing – review and editing (equal). **Yingying Du:** Investigation (equal); resources (equal); validation (equal); writing – review and editing (equal). **Yunmei Liu:** Conceptualization (equal); data curation (equal); formal analysis (equal); investigation (equal); methodology (equal); resources (equal); software (equal); validation (equal); visualization (equal); writing – original draft (equal). **Lei Huang:** Conceptualization (equal); data curation (equal); formal analysis (equal); investigation (equal); methodology (equal); project administration (equal); resources (equal); supervision (equal); validation (equal); visualization (equal); writing – original draft (equal); writing – review and editing (equal). **Guoping Sun:** Conceptualization (equal); funding acquisition (equal); investigation (equal); project administration (equal); resources (equal); supervision (equal); validation (equal); writing – review and editing (equal).

## FUNDING INFORMATION

This work was supported by the National Natural Science Foundation of China (82072751), Shanghai Pujiang Program (21PJ1409700), and Start‐up Fund for the Introduction of High Level Talents by Ruijin Hospital, Shanghai Jiao Tong University School of Medicine. The funders had no involvement in study design; in the collection, analysis, or interpreteation of data; in the writing of the report; or in the decision to submit the paper for publication.

## CONFLICT OF INTEREST STATEMENT

The authors declare that there are no competing interests.

## Supporting information


Appendix S1

## Data Availability

Restrictions apply to the availability of the data for this study, which were used under license, and so are not publicly available.
