# Supplementary material for: Associations of circulating immunomarkers with the efficacy of immunotherapy for primary hepatic carcinoma
Source: Cancer Med. 2023 Dec 6;12(24):21830–48. doi: 10.1002/cam4.6754 (PMC10757102; doi:10.1002/cam4.6754)
Supplement: Supplementary file 1 — Appendix S1 [file CAM4-12-21830-s001.docx]

**Supplementary materials**

**Table S1.** Clinical and pathological characteristics of the included patients with hepatocellular carcinoma (HCC), stratified by neutrophil-to-lymphocyte ratio (NLR)

**Table S2.** Clinical and pathological characteristics of the included patients with hepatocellular carcinoma (HCC), stratified by monocyte-to-lymphocyte ratio (MLR)

**Table S3.** Clinical and pathological characteristics of the included patients with hepatocellular carcinoma (HCC), stratified by platelet-to-lymphocyte ratio (PLR)

**Figure S1.** (**A**) Overall survival (OS) and (**B**) progression-free survival (PFS) in patients with hepatocellular carcinoma and with baseline NLR <5 vs ≥5.

**Table S1.** Clinical and pathological characteristics of the included patients with hepatocellular carcinoma (HCC), stratified by neutrophil-to-lymphocyte ratio (NLR)

| Characteristics (HCC) | NLR<3.38 (n=65) | NLR≥3.38 (n=39) | P value |
| --- | --- | --- | --- |
| Age, >65 | 28 (26.9) | 9 (8.7) | 0.056 |
| Sex, male | 55 (52.9) | 31 (29.8) | 0.594 |
| BMI | 22.70±3.69 | 21.92±3.58 | 1.000 |
| ECOG performance status score |  |  |  |
| 0 | 5 (10.2) | 1 (1.6) | 0.291 |
| 1 | 55 (84.7) | 50 (82.0) |  |
| 2-4 | 5 (5.1) | 10 (16.4) |  |
| Smoking status, current/former | 16 (15.4) | 8 (7.7) | 0.811 |
| Alcohol drinking status, current/former | 10 (9.6) | 5 (4.8) | 0.781 |
| BCLC |  |  | **0.022** |
| 0 or A | 4 (6.2) | 1 (2.6) |  |
| B | 15 (23.1) | 2 (5.1) |  |
| C | 46 (70.8) | 36 (92.3) |  |
| Child-Pugh |  |  | 0.722 |
| A | 1 (0.9) | 2 (1.9) |  |
| B | 63 (60.6) | 37 (35.6) |  |
| C | 1 (0.9) | 0 (0.0) |  |
| Cirrhosis, yes | 9 (8.7) | 5 (10.2) | 1.000 |
| History of hepatitis B, yes | 25 (24.0) | 14 (13.5) | 0.837 |
| HBV-DNA positive, yes | 4 (3.8) | 4 (3.8) | 0.466 |
| Surgical treatment, yes | 29 (27.9) | 19 (18.3) | 0.691 |
| Chemotherapy, yes | 7 (6.7) | 4 (3.8) | 1.000 |
| Molecular targeted therapy, yes | 57 (54.8) | 31 (29.8) | 0.276 |
| Number of Metastatic Sites, ≥2 | 25 (38.5) | 24 (61.5) | **0.027** |
| Presence of extrahepatic metastases, yes | 35 (53.8) | 31 (79.5) | **0.011** |

Statistically significant *P* values are shown in bold.

BMI, body mass index; ECOG, Eastern Cooperative Oncology Group; BCLC, Barcelona Clinic Liver Cancer; NLR, neutrophil-to-lymphocyte ratio; PLR, platelet-to-lymphocyte ratio; MLR, monocyte-to-lymphocyte ratio.

**Table S2.** Clinical and pathological characteristics of the included patients with hepatocellular carcinoma (HCC), stratified by monocyte-to-lymphocyte ratio (MLR)

| Characteristics (HCC) | MLR<0.28 (n=45) | MLR≥0.28 (n=59) | P value |
| --- | --- | --- | --- |
| Age, >65 | 18 (17.3) | 19 (18.3) | 0.418 |
| Sex, male | 41 (39.4) | 45 (43.3) | 0.067 |
| BMI | 21.91±3.40 | 23.06±3.90 | 1.000 |
| ECOG performance status score |  |  | **0.006** |
| 0 | 5 (11.1) | 1 (1.7) |  |
| 1 | 39 (86.7) | 48 (81.4) |  |
| 2-4 | 1 (2.2) | 10 (16.9) |  |
| Smoking status, current/former | 13 (12.5) | 11 (10.6) | 0.247 |
| Alcohol drinking status, current/former | 8 (7.7) | 7 (6.7) | 0.414 |
| BCLC |  |  | **0.007** |
| 0 or A | 2 (4.4) | 3 (5.1) |  |
| B | 13 (28.9) | 4 (6.8) |  |
| C | 30 (66.7) | 52 (88.1) |  |
| Child-Pugh |  |  | 0.256 |
| A | 0 (0.0) | 3 (2.9) |  |
| B | 45 (43.3) | 55 (52.9) |  |
| C | 0 (0.0) | 1 (0.9) |  |
| Cirrhosis, yes | 5 (4.8) | 9 (8.7) | 0.578 |
| History of hepatitis B, yes | 15 (14.4) | 24 (23.1) | 0.541 |
| HBV-DNA positive, yes | 2 (1.9) | 6 (5.8) | 0.466 |
| Surgical treatment, yes | 21 (61.0) | 27 (39.3) | 1.000 |
| Chemotherapy, yes | 6 (5.8) | 5 (4.8) | 0.525 |
| Molecular targeted therapy, yes | 39 (37.5) | 49 (47.1) | 0.785 |
| Number of Metastatic Sites, ≥2 | 19 (18.3) | 30 (28.8) | 0.431 |
| Presence of extrahepatic metastases, yes | 27 (26.0) | 39 (37.5) | 0.544 |

Statistically significant *P* values are shown in bold.

BMI, body mass index; ECOG, Eastern Cooperative Oncology Group; BCLC, Barcelona Clinic Liver Cancer; NLR, neutrophil-to-lymphocyte ratio; PLR, platelet-to-lymphocyte ratio; MLR, monocyte-to-lymphocyte ratio.

**Table S3.** Clinical and pathological characteristics of the included patients with hepatocellular carcinoma (HCC), stratified by platelet-to-lymphocyte ratio (PLR)

| Characteristics (HCC) | PLR<227.18 (n=92) | PLR≥227.18 (n=12) | P value |
| --- | --- | --- | --- |
| Age, >65 | 34 (32.7) | 3 (2.9) | 0.371 |
| Sex, male | 76 (73.1) | 10 (9.6) | 0.694 |
| BMI | 21.17±2.00 | 22.57±3.79 | 0.831 |
| ECOG performance status score |  |  | 0.689 |
| 0 | 6 (5.8) | 0 (0.0) |  |
| 1 | 76 (73.1) | 11 (10.6) |  |
| 2-4 | 9 (8.7) | 2 (1.9) |  |
| Smoking status, current/former | 23 (22.1) | 1 (0.9) | 0.290 |
| Alcohol drinking status, current/former | 15 (14.4) | 0 (0.0) | 0.206 |
| BCLC |  |  | 0.606 |
| 0 or A | 5 (6.8) | 0 (0.0) |  |
| B | 16 (15.4) | 1 (0.9) |  |
| C | 70 (67.3) | 12(11.5) |  |
| Child-Pugh |  |  | 1.000 |
| A | 1 (0.9) | 2 (1.9) |  |
| B | 87 (83.7) | 13 (12.5) |  |
| C | 1 (0.9) | 0 (0.0) |  |
| Cirrhosis, yes | 13 (12.5) | 1 (0.9) | 1.000 |
| History of hepatitis B, yes | 36 (34.6) | 3 (2.9) | 0.362 |
| HBV-DNA positive, yes | 8 (7.7) | 0 (0.0) | 0.592 |
| Surgical treatment, yes | 46 (50.0) | 2 (16.7) | **0.019** |
| Chemotherapy, yes | 8 (7.7) | 3 (2.9) | 0.139 |
| Molecular targeted therapy, yes | 79 (76.0) | 9 (8.7) | 0.113 |
| Number of metastatic sites, ≥2 | 45 (43.3) | 4 (3.8) | 0.246 |
| Presence of extrahepatic metastases, yes | 58 (55.8) | 8 (7.7) | 1.000 |

Statistically significant *P* values are shown in bold.

BMI, body mass index; ECOG, Eastern Cooperative Oncology Group; BCLC, Barcelona Clinic Liver Cancer; NLR, neutrophil-to-lymphocyte ratio; PLR, platelet-to-lymphocyte ratio; MLR, monocyte-to-lymphocyte ratio.


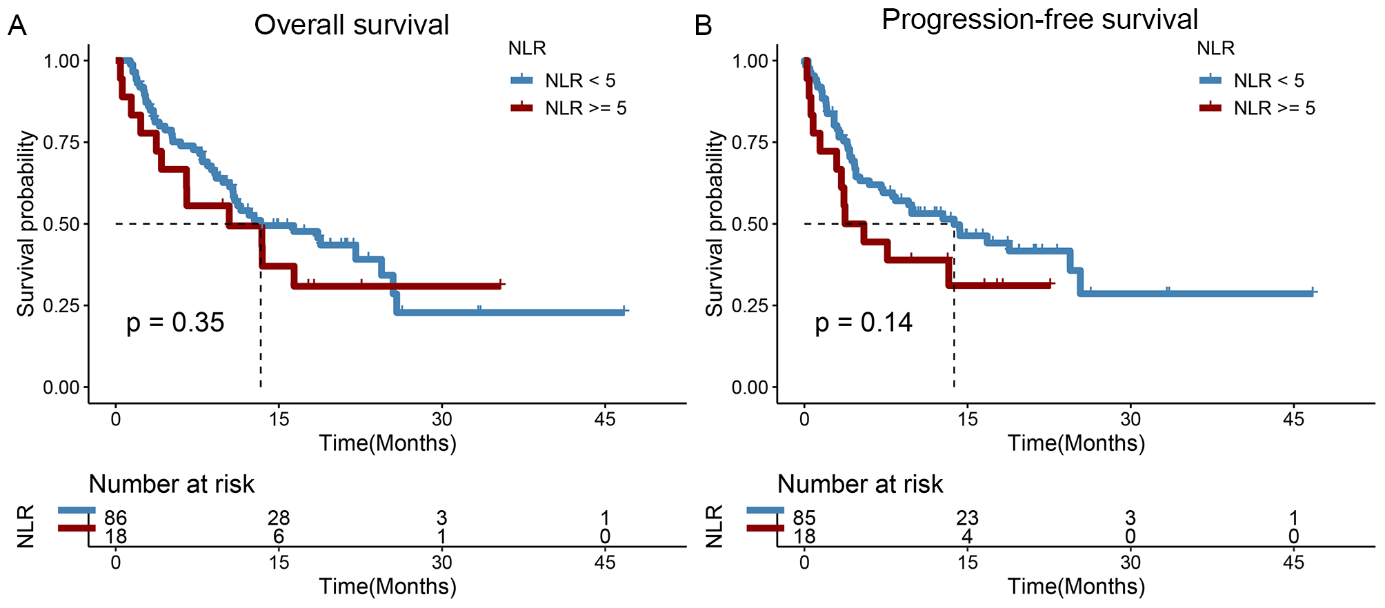


**Figure S1.** (**A**) Overall survival (OS) and (**B**) progression-free survival (PFS) in patients with hepatocellular carcinoma and with baseline NLR <5 vs ≥5.
